# Innovationskrise im staatlichen Theatersektor? Eine Längsschnitt-Analyse für Theater in Nordrhein-Westfalen, 1995–2018

**DOI:** 10.1007/s11577-022-00846-3

**Published:** 2022-07-27

**Authors:** Maria Glasow, Thomas Heinze

**Affiliations:** Institut für Soziologie, Bergische Universität Wuppertal, Gaußstraße 20, 42119 Wuppertal, Deutschland

**Keywords:** Kultursoziologie, Uraufführung, Spielplan, Kulturorganisationen, Organisationsfeld, Cultural Sociology, Premiere, Repertoire, Cultural organization, Organizational field

## Abstract

Der vorliegende Beitrag untersucht die kulturelle Innovationstätigkeit staatlicher deutscher Theater am Beispiel des bevölkerungsreichsten Bundeslandes Nordrhein-Westfalen (NRW). Während bisherige Diagnosen, einschließlich die sozialstrukturell geprägte Publikumsforschung, die Nachfrageseite in den Mittelpunkt stellen und dabei vor allem den stetigen Rückgang und die Alterung des Publikums diagnostizieren, geht unsere Analyse auf die Angebotsseite ein, insbesondere die Zahl von Ur- und Erstaufführungen und ihre Übernahme ins Repertoire. Der Beitrag zeigt, dass die bisherigen Anstrengungen der kommunalen und landeseigenen Theater, die Anzahl der Spielstätten und der dort aufgeführten Stücke zu erhöhen, nicht ausgereicht haben, um den Rückgang des Publikums zu stabilisieren. Gleichzeitig werden nur wenige neue Stücke auf den Spielplan gesetzt, von welchen sich noch weniger langfristig im Repertoire etablieren können. Unseren Ergebnissen zufolge wird der Theatersektor seine Erneuerungsfähigkeit nur sichern können, wenn eine Verschiebung hin zu deutlich mehr neuen Stücken erfolgt, die neues Publikum anziehen. Ein für Erneuerung grundsätzlich günstiger institutioneller Kontext ist der dezentrale Wettbewerb, der den Theatersektor in NRW prägt.

**Zusatzmaterial online:**

Zusätzliche Informationen sind in der Online-Version dieses Artikels (10.1007/s11577-022-00846-3) enthalten.

## Abstract (en)

This paper examines cultural innovations in German public theaters, using North Rhine-Westphalia (NRW) as the most populated region as an example. While existing analyses, including social structure–centered audience research, have focused on the demand side, diagnosing in particular the steady decline and aging of the cultural audience, our analysis addresses the supply side, especially the number of premieres and new productions as well as their adoption into the repertoire. The paper shows that recent efforts by public theaters on the municipal or regional level to increase both the number of venues and the number of plays have not been sufficient to stabilize the declining audience. Too few new plays are scheduled, of which even fewer make it into the long-term repertoire. Our results suggest that theaters can retain their capability for renewal only by staging significantly more new plays, thus attracting new audiences. With regard to such renewal, decentralized competition as a characteristic of the NRW theater landscape seems a favorable institutional context.

## Einleitung

Der vorliegende Beitrag untersucht die Innovationstätigkeit des öffentlich finanzierten Theatersektors. Er versteht sich als Beitrag zur empirisch-analytisch ausgerichteten kultursoziologischen Forschungstradition, die sich mit kultureller Vielfalt und kulturellen Neuerungen beschäftigt. Hierbei sind zwei Forschungspfade zu unterscheiden. Der erste Pfad geht auf DiMaggio und Stenberg (1985a, b) zurück und stellt Nonkonformität des Repertoires in den Mittelpunkt. Auf der anderen Seite stehen Autoren^1^ wie Castañer und Campos (2002), Kremp (2010) oder Gerlach-March (2011), die Neuerungen im Repertoire untersuchen. Die vorliegende Arbeit gehört zum zweiten Forschungspfad, der kulturelle Neuerungen in den Mittelpunkt stellt.

Im ersten Forschungspfad wird Nonkonformität gleichbedeutend mit „Vielfalt“ und „Diversität“ verwendet, wobei damit nicht die Diversität der Kulturschaffenden hinsichtlich ihrer ethnischen sowie regionalen Herkunft oder ihrer sexuellen Identität (z. B. Gerhards et al. 2020; Sharifi 2014) gemeint ist, sondern die Vielfalt der kollektiv erzeugten Kulturprodukte innerhalb organisationaler Felder (Dowd et al. 2002; Durand und Kremp 2016; Heilbrun 2001; Jensen und Kim 2014; Kim und Jensen 2011). Im zweiten Forschungspfad wird unter kulturellen Neuerungen die Verbreitung neuer Autoren (oder Komponisten) und deren Stücken verstanden (Castañer 2014; Gerlach-March 2011; Kremp 2010). Beiden Forschungspfaden liegt ein differenztheoretischer Kulturbegriff zugrunde, der von einem Alltagsverständnis von Kultur ausgeht und Felder wie bildende Kunst, klassische Musik, Literatur sowie Theater und Oper einschließt (vgl. Adloff et al. 2014; Reckwitz 2015).

Die Innovationstätigkeit des Theatersektors in Deutschland wurde bis auf wenige Ausnahmen (Gerlach-March 2011; Neligan 2006) kaum untersucht. Quantitativ-längsschnittliche Analysen fehlen hierzu bislang völlig. Wir stoßen somit in eine Forschungslücke, die wir mit dem vorliegenden Artikel teilweise schließen wollen. Im Fokus dieser Studie stehen öffentlich finanzierte Theater in Nordrhein-Westfalen (NRW). Diese Auswahl lässt sich zum einen damit begründen, dass NRW das bevölkerungsreichste Bundesland ist und die bundesweit höchste räumliche Dichte an Theatern aufweist. Zum anderen fällt hier der Besucherschwund in öffentlichen Theatern im Beobachtungszeitraum (1995–2018) doppelt so hoch aus wie in Deutschland insgesamt, was als ein Krisensignal interpretiert werden kann (vgl. Abschn. 2). Diesbezüglich sind Studien von Reuband (2005, 2013, 2016) aufschlussreich, der mithilfe von Besucherbefragungen in Köln und Düsseldorf (den beiden größten Städten NRWs) zeigen konnte, dass das Opern‑, Theater- und Konzertpublikum immer älter und nicht durch jüngere Kohorten ersetzt wird.

Die Alterung und der damit verbundene langfristige Schwund des Publikums ist verstärkt seit den 2000er-Jahren auch im Feuilleton überregionaler Zeitungen thematisiert worden (z. B. Schindhelm 2000; Schlaffer 2014; Sucher 2001). Hierbei wird eine bereits in den 1980er-Jahren artikulierte Befundlage aufgegriffen, die lautet: „Das Theater stirbt, wenn sein Publikum ausstirbt“ (Henrichs 1984). Interessant ist, dass im Feuilleton eine „Überalterung“ der Theaterkritiker (z. B. Makowsky 2001), Experimentiermüdigkeit bei Inszenierungen und nichtausverkaufte Premieren (z. B. Rossmann 2006; Stadelmaier 2002) erwähnt werden. Zusammen mit der Forderung nach mehr neuen Stücken (z. B. Stephan 2003) lässt sich somit eine doppelte Befundlage im Feuilleton konstatieren, dass nämlich nicht nur das Publikum schwindet, sondern zugleich die Innovationstätigkeit der Theaterbetriebe zu wünschen übrig lässt.

Bislang hat sich die auf Sozialstruktur und Lebensstile fokussierte kultursoziologische Forschung (insbesondere im deutschsprachigen Raum) mit der Frage beschäftigt, wie sozialer Status und kultureller Geschmack zusammenhängen und sich in diesem Zusammenhang mit den gegensätzlichen Thesen der beiden Schlüsselwerke von Bourdieu (1984) und Peterson (1992) beschäftigt (Gerhards et al. 2013; Kunißen et al. 2018; Rössel 2006; Rössel et al. 2017). Zu erwähnen sind diesbezüglich auch umfangreiche Bevölkerungs- und Publikumsumfragen, durch welche ein recht differenziertes Bild über die aktuellen und potenziellen Besucher vorliegt (z. B. Glogner-Pilz und Lutz 2011; Renz 2014; Reuband 2017b; Tauchnitz 2004). In der deutschen Kultursoziologie ist hingegen die Frage, inwiefern sich die kulturelle Praxis selbst erneuern und neue Publikumsschichten erschließen kann, kaum thematisiert worden. Anstatt also immer dieselben Stücke auf die Bühne zu bringen, die immer weniger Nachfrage erzeugen, könnten Theater durch die Aufführung neuer Stücke ihr Repertoire erneuern und sich auf diese Weise mittel- und langfristig neue Publikumsschichten erschließen. Zu genau dieser Überlegung fehlen jedoch empirische Erkenntnisse. Der vorliegende Beitrag untersucht daher, in welchem Umfang neue Stücke inszeniert werden und sich im Repertoire etablieren können.

Bevor der Forschungsstand im dritten Abschnitt eingeführt und zur Hypothesenbildung herangezogen wird, skizziert der folgende zweite Abschnitt die Struktur des Theatersektors in NRW und zieht Vergleiche zur gesamtdeutschen Situation. Im vierten Abschnitt erfolgt eine Beschreibung des Datensatzes und der verwendeten Analysemethoden, dem sich im fünften Abschnitt die Darstellung der empirischen Befunde anschließt. Im sechsten Abschnitt werden die Befunde diskutiert und Schlussfolgerungen gezogen.

## Strukturdaten zum öffentlichen Theatersektor in NRW

Öffentliche Theater sind „stehende, […] spielende Theater und Landesbühnen (Wanderbühnen) mit eigenem Ensemble, jedoch nicht Tourneetheater und Laienbühnen (Märchenbühnen, Heimatbühnen) sowie Varietés und Kabaretts […], deren rechtliche und/oder wirtschaftliche Träger Länder, Gemeinden, Gemeindeverbände sind, unabhängig davon, in welcher Rechtsform sie betrieben werden“ (Deutscher Bühnenverein [DBV] 2019a). Aufgrund des großen Anteils an öffentlichen Theatern weist der deutsche Theatersektor einige Besonderheiten in Bezug auf seine Organisationsstruktur und das System der staatlichen Unterstützung auf. Hier sind zu nennen: eine dezentrale geografische Verbreitung, ein Regiemanagement, Mehrspartentheater sowie eine große Anzahl an Abonnements. Zudem sind die öffentlichen im Vergleich zu den privaten Theatern größer und hierarchischer und es dominieren künstlerische im Gegensatz zu kommerziellen Entscheidungsträgern (Gerlach-March 2011; Neligan 2006). Dementsprechend wird das Modell der Theaterförderung in Deutschland als etatistisch bezeichnet. Sowohl Kultur allgemein als auch Theater im Besonderen sind in Deutschland vorrangig eine Aufgabe der Bundesländer, wobei die öffentliche Theaterfinanzierung ungefähr jeweils zu Hälfte von den Ländern und den Kommunen getragen wird (Abfalter 2010).

Tabelle 1 stellt Kennzahlen zu allen kommunalen und landeseigenen Theatern in NRW seit der Spielzeit 1995/96 (zur Begründung des Analysezeitraums vgl. Abschn. 4) dar; inbegriffen sind auch Mehrspartentheater. Bei Betrachtung der Kennzahlen lässt sich feststellen, dass ihre Anzahl recht konstant geblieben ist (Tab. 1). Weiterhin lässt sich Tab. 1 entnehmen, dass die Anzahl der Spielstätten in NRW von 91 (1995/96) auf 128 (2017/18) ansteigt (plus 41 %), wobei es in der Spielzeit 2010/11 einen Spitzenwert von 165 Spielstätten gab. Allerdings folgt die Zahl der angebotenen Sitzplätze einem umgekehrten Trend: Die pro Spielstätte angebotene Anzahl von Sitzplätzen sinkt von 484 (1995/96) auf 320 (2017/18). Die Angebotsausweitung bei den Spielstätten führt zu einer Reduktion des durchschnittlichen Angebots an Sitzplätzen.

**Table 1:** 

	1995/1996	2000/2001	2005/2006	2010/2011	2015/2016	2017/2018
Anzahl Theaterunternehmen	26	26	25	26	26	25
Anzahl Spielstätten	91	108	134	165	135	128
Anzahl Sitzplätze in Tsd.	44	43	46	49	41	41
Veranstaltungen am Standort	8963	9532	9342	9910	9784	9784
Vollpreis‑/Tageskarten in Tsd.	1034	1100	1024	1131	1200	1077
Abonnements in Tsd.	897	806	689	618	567	573
Besucher am Standort in Tsd.	3617	3415	3190	3107	3016	2864
Reale Betriebseinnahmen	62.734	54.895	54.301	56.680	55.802	54.612
Zuschüsse priv. Einrichtungen	1236	2404	3590	3931	5117	5541
Reale Zuschüsse (Bund und Länder)	366.671	341.801	325.334	324.618	323.505	343.351
Einnahmen insgesamt	436.266	402.727	389.258	400.563	392.289	410.754
Anzahl Theaterunternehmen	22	21	22	22	22	22
Anzahl Spielstätten	85	108	131	165	131	131
Anzahl Aufführungen	5797	5917	5665	5979	6107	5123
Gespielte Stücke insgesamt	361	364	473	508	514	454
Davon: Erst- und Uraufführungen	42	49	38	69	63	58

Quelle: DBV (1997–2019a, 1997–2019b); Finanzangaben in Tsd. Euro und inflationsbereinigt (Basis: 1995)

Der untere Teil der Tabelle bezieht sich ausschließlich auf die *n* = 22 Theater, welche Teil der Analyse der Repertoiresentscheidungen sind (Abschn. 5.1)

Der deutschlandweite Trend ist ähnlich (Tab. A1 im Online-Anhang). Hier sinkt die pro Spielstätte durchschnittlich angebotene Anzahl von Sitzplätzen von 369 (1995/96) auf 317 (2017/18). Es gibt somit eine Entwicklung hin zu vielen unterschiedlichen Spielstätten je Theater (in NRW von durchschnittlich 3,5 in 1995/96 auf 5,1 Spielstätten in 2017/18 pro Theater), welche aber weniger Sitzplätze zur Verfügung haben und damit auch leichter zu füllen sind. Dadurch sind Theater in der Lage, mehr unterschiedliche Stücke gleichzeitig aufführen zu lassen. Da zudem die Sitzplatzkapazitäten der einzelnen Spielstätten vergleichsweise gering sind, sind „Misserfolge“, also aufgeführte Stücke mit nur wenigen Besuchern, besser auszugleichen.

So wie die Anzahl der angebotenen Sitzplätze sinkt auch die Anzahl der theatereigenen Veranstaltungen trotz steigender Anzahl von Spielstätten (Tab. 1). In NRW dünnt die durchschnittliche Anzahl von Veranstaltungen pro Spielstätte linear von 98 (1995/96) auf 77 (2017/18) aus. Dies bedeutet, dass trotz der in 2017/18 gestiegenen Anzahl von Spielstätten (plus 37) dort im Mittel knapp ein Viertel weniger Veranstaltungen als in 1995/96 aufgeführt wurden (minus 22). Der Trend im deutschlandweiten Vergleich ist zwar gleichförmig, aber weniger stark (Tab. A1). Insgesamt dünnt die durchschnittliche Anzahl von Veranstaltungen pro Spielstätte deutschlandweit von 95 (1995/96) auf 79 (2017/18) aus. Ebenso ist ein Rückgang bei der Aufführungshäufigkeit von Stücken feststellbar. Bei den 22 Theatern, für die verlässliche Daten zu ihren Werken vorliegen, sinkt die durchschnittliche Zahl der Aufführungen pro Theaterstück im Beobachtungszeitraum von 16 auf 11 (Tab. 1, unterer Teil).

Die Anzahl der Besucher folgt der sinkenden Anzahl theatereigener Veranstaltungen. So sinkt in NRW die Besucherzahl von 3,61 Mio. (1995/96) auf 2,86 Mio. (2017/18) – ein Minus von 20,8 %. Besonders dramatisch ist der Rückgang bei den Abonnements, die um 36 % zurückgehen. Diese Entwicklung konnte nicht durch die Vollpreis- oder Tageskarten aufgefangen werden, da sich ihre Anzahl nur um 4 % erhöht. Die Zahlen machen deutlich, dass zum einen eine leichte Verschiebung der Abonnements hin zu Einzelticketverkäufen stattfindet und zum anderen, dass der Besucherrückgang vor allem durch den Rückgang der Abonnements bedingt ist. Dies bedeutet, dass der feste Publikumsstamm rückläufig ist. Möglicherweise möchten sich Besucher nicht mehr mit einem Abonnement festlegen, sondern wollen spontan Einzeltickets für bestimmte Aufführungen kaufen, die sie interessieren.

Publikumsbefragungen identifizieren einen weiteren Grund für die rückläufige Zahl von Besuchern. So schließen vor allem ältere Personen Abonnements ab (Reuband 2007, 2017a), welche jedoch nach und nach versterben und nicht in gleichem Umfang durch jüngere Personen ersetzt werden. Auch der deutschlandweite Trend bei den Besuchern ist von Schrumpfung gekennzeichnet. Hier ist ein Absinken von 20,55 Mio. (1995/96) auf 18,53 Mio. Besucher (2017/18) zu konstatieren – minus 9,8 % (Tab. A1). Anders als die Besucherzahlen steigt im Zeitraum 1995/96–2017/18 die Anzahl der gespielten Stücke insgesamt (plus 38,1 %) und der Erst‑/Uraufführungen (plus 25,8 %) über den beobachteten Zeitraum von 361 bzw. 42 (1995/96) auf 454 bzw. 58 (2017/18) an. Hierbei könnte der Eindruck entstehen, dass ein Zusammenhang zwischen den sinkenden Besucherzahlen in NRW und den steigenden Erst‑/Uraufführungszahlen oder den Stücken insgesamt existiert. Dies ist jedoch nicht der Fall: Es lassen sich keine statistisch signifikanten Korrelationen (*p* < 0,05) nachweisen.

Der Rückgang der Besucher in NRW macht sich auch bei den inflationsbereinigten Gesamteinnahmen der kommunalen und landeseigenen Theater bemerkbar. Diese sinken im Zeitraum um 5,8 %, wobei dieser Rückgang bei den Betriebseinnahmen höher (minus 13 %) als bei den Subventionen (minus 6,4 %) ausfällt. Im Gegensatz zu NRW zeigt sich bundesweit kein Rückgang bei den Finanzen: die Höhe der inflationsbereinigten Gesamteinnahmen der Theater bleibt praktisch konstant, wobei der wohl deutlichste Unterschied zu NRW in der erheblichen Steigerung der Betriebseinnahmen liegt (plus 20 %), während die Subventionen nur geringfügig sinken (minus 2 %) und Zuschüsse privater Einrichtungen – wie in NRW – eine ähnliche Größenordnung für die Gesamteinnahmen aufweisen (1,2 %).

Zieht man die bisherigen strukturellen Befunde für NRW (und Deutschland) zusammen, dann lässt sich eine Nachfragekrise ausmachen: Trotz einer steigenden Anzahl von Stücken sinkt die Zahl der Besucher. Dieser Befund stimmt mit der Aussage von Haselbach et al. (2012) überein, die eine „kulturelle Expansion“ seit Ende der 1970er-Jahre feststellen, welche eine Erhöhung des Angebots bewirkte. Allerdings werden mit dieser Feststellung nicht die Fragen beantwortet, ob erstens die kulturelle Expansion des Theatersektors auch mit der Erneuerung des Theaterprogramms im Sinne von Erst- und Uraufführungen einhergeht und zweitens diese Expansion ausreicht, um neues Publikum zu gewinnen. Die Antworten auf diese beiden Fragen sind eng mit der Kulturpolitik verknüpft, die die Finanzierung von Theatern aus Steuermitteln gegenüber der Öffentlichkeit legitimieren muss (Zimmer und Mandel 2021). Theater sind in eine institutionelle Umwelt eingebettet, zu der neben den ökonomischen, rechtlichen und politischen Rahmenbedingungen auch das Angebot und die Nachfrage auf dem Freizeit‑, Medien- und Unterhaltungsmarkt (Kinos, Fernsehen, Streaming, Games) gehören sowie der damit verbundene Konkurrenzdruck um die Gewinnung sowie Bindung von Besuchern (Glogner-Pilz und Föhl 2011; Wagner 2005). Bevor diese Frage im fünften Abschnitt empirisch untersucht wird, folgen zunächst eine theoretische Einordnung und die Hypothesenbildung.

## Theoretische Einordnung und Hypothesenbildung

Wie in der Einleitung erwähnt, existieren in der kultursoziologischen Literatur zwei Forschungspfade, die auf unterschiedliche Weise die Arbeitsprodukte kultureller Einrichtungen wie Theater, Konzert- und Opernhäuser sowie Museen konzeptualisieren. Der erste Pfad geht auf DiMaggio und Stenberg (1985a, b) zurück und stellt Nonkonformität des Repertoires in den Mittelpunkt. Auf der anderen Seite stehen Autoren wie Castañer und Campos (2002), Kremp (2010) oder Gerlach-March (2011), die Neuerungen im Repertoire, also die Verbreitung neuer Autoren oder Komponisten und deren Stücke analysieren.

DiMaggio und Stenberg (1985a, b) gehen davon aus, dass Innovativität bei Theatern nicht direkt gemessen werden kann, weil sich kein Konsens darüber herstellen lasse, was innovativ sei: Konkrete Stücke, Autoren oder Theater lassen sich nur schwer auf einer ordinalen Skala verorten. Daher analysieren sie den Grad der Abweichung des Repertoires eines ausgewählten Theaters vom Repertoire aller anderen Theater. Je mehr das Repertoire eines Theaters mit dem der anderen Theater übereinstimmt, umso konformer, und: je stärker das Repertoire des untersuchten Theaters von dem der übrigen Theater abweicht, desto nonkonformer sei die Aufführungspraxis. Konformität wird somit als Abweichung einer Kulturorganisation von ihrem Organisationsfeld operationalisiert. Die Abweichung findet bei DiMaggio und Stenberg (1985a, b) Ausdruck im Konformitätsindex, der in Folgestudien entweder direkt (Jensen und Kim 2014; Kim und Jensen 2011; Neligan 2006; O’Hagan und Neligan 2005; Tamburri et al. 2015) oder in abgewandelter Form verwendet wurde (Dowd et al. 2002; Durand und Kremp 2016; Pompe et al. 2011).

Zu den Ergebnissen der genannten Studien zählt beispielsweise, dass die Konformität des Repertoires mit zunehmender Abhängigkeit vom Markt und verfügbaren Sitzplätzen zunimmt, während der Zugang zu potenziellen Zuschauern mit hohem kulturellen Kapital die Konformität senkt (DiMaggio und Stenberg 1985b; Neligan 2006; O’Hagan und Neligan 2005; Tamburri et al. 2015). Demgegenüber führt ein heterogenes Publikum mit unterschiedlichen Präferenzen eher zu einer Ausbalancierung zwischen konventionellen und unkonventionellen Stücken im Programm von Theatern (Jensen und Kim 2014).

Der Konformitätsindex wurde in der Literatur dafür kritisiert, dass er von der Vorstellung ausgeht, dass eine nonkonforme Aufführungspraxis als solche schon innovativ sei. Nonkonformität, so die Kritik, sei keineswegs mit der Aufführung neuer, bislang unbekannter Komponisten oder Autoren und deren Stücken gleichzusetzen (Castañer und Campos 2002). Im Gegensatz zu der an DiMaggio und Stenberg (1985a, b) orientierten Definition schlagen Castañer und Campos (2002) vor, den in der Wissenschafts- und Technikforschung sowie der Kreativitätsforschung üblichen Innovationsbegriff zu benutzen, bei dem die Herstellung und Verbreitung neuen Wissens und neuer Artefakte im Mittelpunkt stehen. Dieser Konzeptualisierung folgt beispielsweise Kremp (2010), der für den Bereich der Sinfonieorchester die Aufführung neuer Komponisten in den Mittelpunkt seiner Analyse stellt.

Weiterhin unterscheidet Gerlach-March (2011) für den Bereich von Theaterproduktionen auf einer Ordinalskala vier Ausprägungen von „Innovativität“: Neuinterpretationen eines Repertoirestückes (= Neuinszenierungen); Interpretationen und Überarbeitungen von Texten oder Materialien für ein Theaterstück aus einem anderen Medium, wie Büchern oder Filmen (= Adaptionen); Übersetzungen oder Übertragungen und Anpassungen eines Stückes aus einer anderen Sprache (= Erstaufführung); und originär neue Stücke (= Uraufführungen). Der Autorin zufolge sind Ur- und Erstaufführungen am innovativsten (Gerlach-March 2011). Es lässt sich feststellen, dass Diversität (oder Vielfalt, Gegenteil: Konformität) und Innovativität (oder Neuerung, Gegenteil: Kanon) nicht direkt gleichzusetzen sind. Diversität und Innovativität sind jedoch einschlägige Konzepte aus der kultursoziologischen Forschung. Mit unserem Datensatz analysieren wir Neuerungen (in Form von Ur- und Erstaufführungen) sowie deren Erfolg im Organisationsfeld (= Innovationen), während wir keine Aussagen zur Konformität machen.

Die von DiMaggio und Stenberg (1985a, b) begründete Tradition hat die empirische Forschung zu kulturellen Innovationen in den letzten Jahrzehnten stark geprägt (Otte 2017). Allerdings zeigen die Überlegungen von Castañer und Campos (2002) sowie die empirischen Untersuchungen von Kremp (2010) und Gerlach-March (2011) einen interessanten Forschungspfad auf, der die bisherigen Studienergebnisse in der Tradition von DiMaggio und Stenberg (1985a, b) sinnvoll ergänzt und zugleich an andere sozialwissenschaftliche Forschungsgebiete (z. B. die Wissenschafts- und Technikforschung sowie die Kreativitätsforschung) anschlussfähig macht (Castañer 2014).

Im Folgenden werden auf der Basis von bisherigen Studien beider Forschungspfade Hypothesen formuliert, die bislang für unterschiedliche Kulturorganisationen empirisch geprüft wurden, insbesondere Theater sowie Opern- und Konzerthäuser außerhalb Deutschlands, vorrangig in den Vereinigten Staaten und dem Vereinigten Königreich (vgl. Castañer 2014). Zum einen sollen entsprechende Zusammenhänge für die sich in öffentlicher Trägerschaft befindlichen Theater in NRW für den Zeitraum 1995–2018 untersucht werden. Zum anderen gehen wir bei der Hypothesenprüfung forschungspragmatisch vor und orientieren uns an den verfügbaren Daten, die größtenteils vom Deutschen Bühnenverein (DBV) stammen (vgl. Abschn. 4).

Die Literatur der beiden erwähnten Pfade der empirischen Kulturforschung bezieht in der Regel zwei Analyseebenen ein, welche auf die neo-institutionalistische Organisationstheorie zurückgehen (DiMaggio und Powell 1983; Meyer und Rowan 1977; Powell und DiMaggio 1991): die Organisationsebene (das jeweilige Theater) und die Ebene des Organisationsfeldes (alle untersuchten Theater), in das die jeweilige Organisation eingebettet ist. Wir orientieren uns ebenfalls an diesen beiden Analyseebenen. Nachfolgend wird der Begriff „Spielplan“ verwendet, um gespielte Stücke eines Theaters in einem Jahr zu beschreiben, während der Begriff „Repertoire“ die gespielten Stücke aller untersuchten Theater über alle Jahre hinweg meint.

Wir testen vier Hypothesen zu Neuerungen und eine Hypothese zu Innovationen. Der Schwerpunkt liegt auf den Neuerungen (Ur- und Erstaufführungen), da diese selbst den Innovationen vorausgehen: Nur wenn eine Neuerung von mindestens einem anderen Theater aufgegriffen wird und sich somit im Repertoire etablieren kann, ist sie eine Innovation.

Die erste Hypothese (*H 1*) folgt der Überlegung, dass sich die Aufführungskapazität von Theatern positiv auf die Zahl der von ihnen gespielten Neuerungen auswirkt. Dowd et al. (2002) präsentieren diesbezüglich den empirischen Befund, dass je mehr US-amerikanische Orchester es gibt und je mehr Konzerte (= Aufführungskapazität) diese geben, die Anzahl neuer Komponisten im Repertoire umso höher ist. Dieser Befund der Feldebene lässt sich nach Kremp (2010) auch auf der Organisationsebene feststellen: Mit steigender Konzertanzahl erhöht sich die Anzahl neuer Komponisten auf den Spielplänen konkreter Orchester. Dieser für Sinfonieorchester untersuchte Zusammenhang zwischen Aufführungskapazitäten und Innovationstätigkeit soll hier auf Theater übertragen werden (*H 1*).

### H 1

Je höher die Zahl der Aufführungen eines Theaters, umso höher ist die Zahl von Neuerungen auf seinem Spielplan.

Weiterhin formulieren wir Hypothesen zur Ressourcensituation von Theatern. Mit der Aufführung neuer Stücke gehen Theater das Risiko eines Misserfolgs ein (Pierce 2000; Sgourev 2012). Je mehr Theater von eigenen Betriebseinnahmen abhängig sind, umso mehr müssen sie auf altbewährte Theaterstücke setzen, um nicht weiteres Publikum zu verlieren. Es liegt daher die Annahme nahe, dass die Abhängigkeit von Betriebseinnahmen die Experimentierfreude senkt. Daher soll überprüft werden, ob der Umfang von eigenen Betriebseinnahmen in Form von Abonnements die Innovationstätigkeit eines Theaters absenkt. Diese Annahme wird von Voss et al. (2006) unterstützt, die herausfanden, dass sich die Zahl der Abonnements ab einem gewissen Punkt negativ auf die Innovationstätigkeit auswirkt. Daher vermuten auch wir, dass je mehr sich das Theaterpublikum an fest gebuchten Tickets orientiert, umso weniger neue Stücke auf dem Spielplan stehen (*H 2*). Umgekehrt können staatliche Subventionen als Puffer für kommerzielle Misserfolge angesehen werden, die die Abhängigkeit von eigenen Betriebseinnahmen verringern (DiMaggio und Stenberg 1985b; O’Hagan und Neligan 2005). Daher sollte die Höhe der staatlichen Subventionen die Experimentierfreude und damit auch die Innovationstätigkeit der Theater erhöhen (*H 3*).

### H 2

Je höher die Anzahl der Abonnements, desto weniger Neuerungen finden sich im Spielplan eines Theaters.

### H 3

Je höher die staatlichen Subventionen, desto mehr Neuerungen finden sich im Spielplan eines Theaters.

Neben der Organisationsebene formulieren wir auch Hypothesen zum Organisationsfeld, in das die Theater eingebettet sind. Diesbezüglich wird der einschlägigen Literatur zufolge die Innovativität des Spielplans vom Wettbewerb beeinflusst. So finden Jensen und Kim (2014) heraus, dass Opernunternehmen ihr Programm vor allem dann effektiv an verschiedene Publikumsgeschmäcker anpassen, wenn wenig Wettbewerbsdruck herrscht. Diesen messen sie anhand eines Index, der die Anzahl unterschiedlicher Stücke im Spielplan eines Theaters mit der räumlichen Dichte von Theatern im räumlichen Umfeld verknüpft. Demzufolge sei es für ein Theater einfacher, Publikum zu gewinnen und zu binden, wenn im weiteren Umkreis nur wenige andere Theater existieren. Zugleich steigt mit zunehmendem Wettbewerbsdruck die Konventionalität des Repertoires eines Theaters (*H 4*). Wir knüpfen an diesen Befund an und untersuchen, ob er sich auf den NRW-Theatersektor und auf die Anzahl der Neuerungen übertragen lässt. Als zusätzliche Variable zur Messung des Wettbewerbsdrucks haben wir die Anzahl der Privattheater in der jeweiligen Stadt mit in unsere Analyse aufgenommen.

### H 4

Je höher der Wettbewerbsdruck, desto weniger Neuerungen werden in den Spielplan des Theaters aufgenommen.

Zusätzlich zur bisherigen abhängigen Variable (AV1), welche die Neuerungen (Ur- und Erstaufführungen) abbildet, untersuchen wir, ob sich die Neuerungen im zeitlichen Verlauf durchsetzen. Dabei haben wir in Anlehnung an Kremp (2010) mithilfe der binären Variable Innovationen (AV2) berechnet, ob ein neues Stück in den zehn Folgejahren nochmals in mindestens einem weiteren der untersuchten NRW-Theater aufgeführt wurde (vgl. Abschn. 4). Unsere Hypothese lautet, dass die Zahl der Neuerungen (Stücke) im Organisationsfeld der NRW-Theater die Herausbildung von Innovationen an einzelnen Theatern befördert (*H 5*). Diesbezüglich findet Kremp (2010) heraus, dass der Erfolg neuer Komponisten positiv mit der Dichte neuer Komponisten im Feld der Orchester verbunden ist. Die Dichte neuer Komponisten steht für Offenheit und Ressourcen für neue Musik auf der Feldebene, durch die die Etablierung neuer Stücke bei konkreten Orchestern befördert wird. Hierbei ist die Vorstellung aus der Populationsökologie leitend (Woywode und Beck 2014), dass ein Organisationsfeld mit hoher Aufnahmekapazität für Neuerungen einen geeigneten Kontext für die Übernahme solcher Neuerungen in die Spielpläne anderer Theater bietet.

### H 5

Je höher die Zahl der Neuerungen (Stücke) in allen Theatern, umso höher ist die Zahl von Innovationen auf dem Spielplan eines Theaters.

## Daten und Methoden

Die Datengrundlage des vorliegenden Beitrags wird jeweils durch die jährlich erscheinenden Theater- und Werkstatistiken des Deutschen Bühnenvereins (1995/96–2017/18) gebildet. Die Werkstatistiken liegen in disaggregierter Form (Organisationsebene) ab 1995/96 vor, sodass erst ab dieser Spielzeit Analysen auf dieser Ebene möglich sind. Den Werkstatistiken ist zu entnehmen, welche Stücke die Theater in der jeweiligen Spielzeit gespielt haben, einschließlich Angaben zu Ur- oder Erstaufführungen sowie Aufführungs- und Besucherzahlen. Die Theaterstatistiken enthalten Angaben zu den Veranstaltungen, Besucherzahlen, Personal, Einnahmen und Ausgaben. Abgebildet sind alle sich in öffentlicher (zumeist kommunaler) als auch einige sich in privater Trägerschaft befindliche Theater. Die Meldung der statistischen Daten der privaten Theater erfolgt, anders als bei öffentlichen Theatern, auf freiwilliger Basis, weshalb sie weit weniger verlässlich in den Theater- und Werkstatistiken abgedeckt sind, was zu erheblichen Datenlücken führt. Zum einen sind die Daten einzelner Privattheater über die einzelnen Jahre unvollständig und zum anderen sind nicht alle Privattheater abgebildet. Ein flächendeckender Vergleich zwischen öffentlichen und privaten Theatern ist also aufgrund der erheblichen Datenlücken mit den Daten des Deutschen Bühnenvereins nicht möglich.

Die Theaterstatistiken des Deutschen Bühnenvereins listen 26 in öffentlicher Hand befindliche Theater in NRW (siehe Tab. 1), von denen zunächst nur diejenigen erhoben wurden, welche Veranstaltungen in der Sparte Schauspiel anbieten (*n* = 22), weshalb das Aachener Puppentheater, die Deutsche Oper am Rhein Düsseldorf, das Hänneschen Theater Köln und das Tanztheater Pina Bausch Wuppertal unberücksichtigt blieben. Von den 22 verbliebenen Theatern wurden diejenigen ausgewählt, die für den betrachteten Zeitraum von 1995–2018 jährlich durchgehend Werte in den abhängigen (AV1 und AV2) und unabhängigen Variablen (UV) aufweisen. Das Mühlheimer Theater an der Ruhr und das Gelsenkirchener Musiktheater im Revier konnten aufgrund erheblicher Datenlücken bei der Regressionsanalyse (*n* = 20) nicht berücksichtigt werden. Allerdings konnten alle *n* = 22 Theater in die Analyse von Repertoiresentscheidungen aufgenommen werden. Daher handelt es sich um eine Vollerhebung aller öffentlichen Theater in NRW, die in der Sparte Schauspiel Veranstaltungen verzeichnen.

Für die vorliegende Analyse mussten die schriftlich verfügbaren Statistiken manuell transkribiert werden, um sie anschließend in Excel und STATA 16 analysieren zu können. Auf dieser Grundlage wurde ein Datensatz aufgebaut, der sowohl für die Analyse von Repertoiresentscheidungen als auch für die Regressionsanalysen zugrunde gelegt wurde.

Die abhängigen und unabhängigen Variablen sowie die Kontrollvariablen unserer Analyse haben wir im Sinne einer Anschlussfähigkeit an die einschlägige Forschungsliteratur und den darin enthaltenen Theorien, Hypothesen sowie Variablen abgeleitet (Abschn. 3). Hierbei wurden zwei abhängige Variablen konstruiert. Die erste abhängige Variable misst die Anzahl von Ur- und Erstaufführungen (Neuerungen) jedes Theaters in der jeweiligen Spielzeit. Die AV1 wird von den Werkstatistiken definiert, sie ist somit eine Information des Deutschen Bühnenvereins. Die zweite abhängige Variable (AV2) misst den Erfolg von Ur- und Erstaufführungen im zeitlichen Verlauf (wiederaufgeführte Neuerungen = Innovationen). Hierzu wurde berechnet, ob eine Ur- oder Erstaufführung in den zehn Folgejahren in mindestens einem weiteren der untersuchten NRW-Theater aufgeführt wurde. Um Verzerrungen der AV2 zu vermeiden, wurden symmetrische Beobachtungszeiträume von jeweils zehn Jahren untersucht. Es fließen in die AV2 somit nur diejenigen Ur- oder Erstaufführungen und unabhängigen Variablen der Spielzeiten 1995/96–2007/08 ein, für die zehn weitere Beobachtungsjahre zur Verfügung stehen. Unsere Datenstruktur sieht daher folgendermaßen aus. Die einzelnen Variablen liegen jährlich pro Theater vor. Bei der Analyse der Neuerungen (AV1) betrachten wir den gesamten Erhebungszeitraum (1995/96–2017/18) und bei der Analyse der Innovationen (AV2) nur die Variablen aus den Jahren 1995/96–2007/08, also nur einen Ausschnitt unseres Gesamtdatensatzes.

Folgende unabhängige Variablen wurden erhoben: Aus den Theaterstatistiken wurde die Anzahl von Abonnements (*H 2*), die Anzahl der Privattheater (*H 4*) sowie die Höhe der staatlichen Subventionen (*H 3*) ermittelt. Aus den Werkstatistiken wurden die Aufführungszahlen (*H 1*) sowie der Competitive-Pressure-Index (CPI) für den öffentlichen Theatersektor (*H 4*) berechnet. Außerdem fungiert die AV1 in einer der Regressionsanalysen als unabhängige Variable (*H 5*). Die Finanzdaten sind inflationsbereinigt (Basis: 1995) und in Euro angegeben. Die Anzahl der Stücke (Werkstatistik) und Spielstätten (Theaterstatistik) fungieren als Kontrollvariablen. Wir interpretieren auch die Kontrollvariablen, allerdings sollte hierbei beachtet werden, dass sie nicht durch Hypothesen belegt sind. Für den CPI wurden die auf Luftlinien basierenden Distanzen in Meilen zwischen den einzelnen Theatern genutzt. Der CPI lautet wie folgt:

$${\sum }_{j=1}^{n}R_{j}{d}_{ij}^{-\alpha }$$

Dabei steht *n* für die Anzahl der Theaterunternehmen im Datensatz, *Rj *steht für die Anzahl der unterschiedlichen Theaterstücke des untersuchten Theaterunternehmens im Untersuchungszeitraum und *d*_*ij*_ für die Distanz in 100 Meilen zwischen dem untersuchten Theaterbetrieb und allen anderen im Datensatz. Für α nutzen wir den Wert 2, da wir davon ausgehen, dass der Wettbewerbsdruck mit zunehmender Entfernung nachlässt. Wie bei Jensen und Kim (2014) hat das Einsetzen der Werte α = 1 und α = 3 in unserer Analyse zu vergleichbaren Ergebnissen geführt. Ebenfalls zu erwähnen ist, dass der CPI den Wettbewerbsdruck hinsichtlich des öffentlichen Theatersektors in NRW abbildet und Privattheater bei der Berechnung des CPI (aufgrund genannter unzureichender Datenlagen) nicht berücksichtigt werden.

## Empirische Ergebnisse

### Häufigkeit von Neuerungen und Innovationen

**Figure 1:**
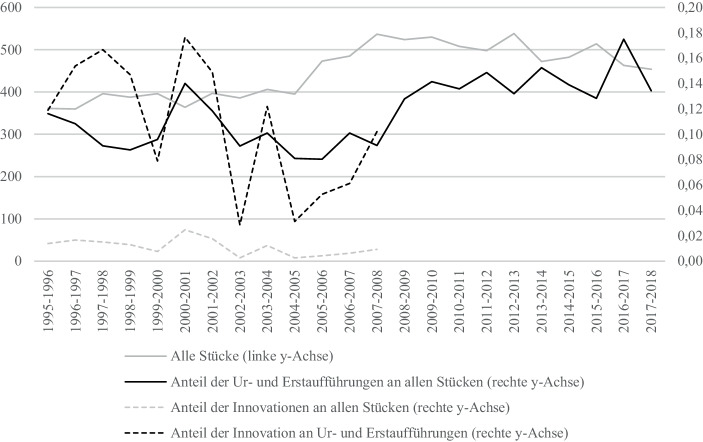

Nachfolgend soll das Repertoire der 22 Theater unseres Datensatzes für den Analysezeitraum (1995/96–2017/18) näher betrachtet werden: So ist die Anzahl aller Aufführungen pro Jahr von *n* = 5797 auf 5123 (−11,6 %) gesunken. Demgegenüber ist die Anzahl der mindestens einmal aufgeführten Theaterstücke von *n* = 361 bis auf *n* = 454 (um 25,8 %) und die Zahl der ur- oder erstaufgeführten Stücke von *n* = 42 auf *n* = 58 (um 34,9 %) gestiegen. Aus diesen Daten lassen sich einige deskriptive Befunde hinsichtlich der Häufigkeit von Neuerungen im Repertoire auf der Ebene des Organisationsfeldes aller NRW-Theater formulieren (AV1). Erstens ist die durchschnittliche Anzahl der Aufführungen eines Stückes im Analysezeitraum von 16 auf 11 gesunken. Einstudierte Stücke werden im Zeitverlauf also in abnehmender Häufigkeit zur Aufführung gebracht. In Übereinstimmung mit diesem Befund hat zweitens die Zahl unterschiedlicher Stücke und die Zahl neuer Stücke zugelegt. Im Zeitverlauf wird drittens sichtbar, dass die Zunahme verschiedener und neuer Stücke auf eine Trendwende Mitte der 2000er-Jahre zurückzuführen ist (Abb. 1). Lag die Anzahl unterschiedlicher (oder neuer) Theaterstücke zwischen 1995/96 und 2006/07 bei durchschnittlich *n* = 393 (bzw. 39), so lag sie in den Jahren 2006/07–2017/18 bei *n* = 500 (bzw. 64). Diese Trendwende wirft die Frage auf, wie sie hinsichtlich des künstlerischen und wirtschaftlichen Erfolgs des öffentlichen Theatersektors in NRW zu interpretieren ist.

Abbildung 1 zeigt, dass ab der Spielzeit 2005/06 die Anzahl der Stücke insgesamt auf jährlich mehr als 500 ansteigt, nachdem der Kurvenverlauf (mit Ausnahme von 2000/01) relativ konstant knapp unter 400 lag. Auch der zugehörige Kurvenverlauf der Neuerungen zeigt einen substanziellen Anstieg Ende der 2000er-Jahre um mehrere Prozentpunkte auf ein neues Plateau, das aber allmählich wieder abflacht. Beide Kurvenverläufe hängen zusammen und verlaufen ähnlich: Von den erfassten 10.327 Theaterstücken (über alle Spielzeiten hinweg summiert, inklusive Mehrfachaufführungen) sind 1195 Erst- und Uraufführungen, was 11,6 % entspricht. Die Prozentzahlen weichen über die Jahre nur geringfügig von diesem Durchschnittswert ab, woraus sich ableiten lässt, dass die Anzahl der Erst- und Uraufführungen von der Anzahl der Theaterstücke abhängt.

Eine Korrelationsanalyse (Pearson) untermauert diese Vermutung. Mit r = 0,731 besteht ein hoher positiver linearer Zusammenhang zwischen der Anzahl der unterschiedlichen, zur Aufführung gebrachten Theaterstücke (nicht zu verwechseln mit Aufführungen) und der Anzahl der Ur- oder Erstaufführungen. Es werden also besonders viele Neuerungen in denjenigen Jahren gespielt, in denen auch insgesamt viele verschiedene Theaterstücke gespielt werden.

Ebenfalls betrachtet werden soll der Kurvenverlauf des Anteils der Innovationen an allen Stücken sowie der Kurvenverlauf der Innovationen an den Ur- und Erstaufführungen. Zu den Innovationen liegen aufgrund des benötigten Berechnungszeitraums von zehn Folgejahren nur Werte für 1995/96–2007/08 vor (für die Berechnung der Innovationen vgl. Abschn. 4). Hierbei wird deutlich, dass der Kurvenverlauf des Anteils der Innovationen an allen Stücken sowie mit den beiden zuvor erläuterten Kurvenverläufen übereinstimmt. Dies spiegelt sich ebenfalls in einem hohen Korrelationswert zwischen der Anzahl der Erst- und Uraufführungen und der Anzahl der Innovationen wider (r = 0,573): Je höher die Anzahl der Neuerungen, desto höher ist auch die Anzahl der Innovationen. Jedoch erreicht der Anteil der Innovationen nur geringe Prozentwerte: Bis auf die Spielzeit 2000/01 liegen alle Anteilswerte unter 2 %. Dies deckt sich mit dem Befund von Gerlach-March (2011) für den Zeitraum 2000/01–2002/03, dass neue Stücke nicht so oft und lange gespielt werden wie bereits etablierte.

Wie in Tab. A2 im Online-Anhang dokumentiert, spielen im Zeitraum 1996/96–2017/18, absolut gesehen, besonders viele neue Stücke das Düsseldorfer Schauspielhaus (*n* = 140), die Bühnen der Stadt Köln (*n* = 128) und das Dortmunder Theater (*n* = 126). Mit etwas Abstand folgt das Bonner Theater (*n* = 90). Schlusslichter sind das Landestheater Dinslaken (*n* = 18), das Theater in Mülheim an der Ruhr (*n* = 11) und das Gelsenkirchener Musiktheater (*n* = 7). Betrachtet man die Anteile der Neuerungen an allen Aufführungen der jeweiligen Theater, zeigt sich hingegen ein differenzierteres Bild. Zwar erreichen Köln (20 %) und Dortmund (18 %) auch hier hohe Werte, aber Gelsenkirchen belegt nunmehr den sechsten Platz (14 %). Die in Tab. A2 gezeigte Gesamtübersicht über die absoluten und relativen Verteilungen der Neuerungen sowie die sich daraus ergebenden Rangplätze bildet die Basis für die Berechnung des Rangkorrelationskoeffizienten nach Spearman. Zwischen beiden Rängen lässt sich ein mittelstarker positiver Zusammenhang (r = 0,578) feststellen. Dies bedeutet, dass je höher die absolute Anzahl an Neuerungen ist, desto höher ist auch der Anteil der Neuerungen an allen Stücken.

Die Frage, ob sich neue Stücke im Repertoire nordrhein-westfälischer Theater etablieren können (AV2), soll mithilfe einer einfachen Berechnung zunächst deskriptiv untersucht werden. Dafür wurde ermittelt, wann die Ur- oder Erstaufführung eines Stückes im Zeitraum 1995/96–2007/08 verzeichnet wurde, um anschließend in den nachfolgenden zehn Spielzeiten zu ermitteln, ob das jeweilige Theaterstück erneut gespielt wird. Für die Stücke, welche in der Spielzeit 1995/96 als Ur- oder Erstaufführung verzeichnet sind, bedeutet dies, dass die Spielzeiten 1996/97–2005/06 überprüft wurden; für 1996/97 die Spielzeiten 1997/98–2006/07, für 1997/98 die Spielzeiten 1998/99–2007/08 usw. Anschließend wurde die ermittelte Zahl in Relation zur Anzahl aller Spielzeiten gesetzt, in denen das Theaterstück hätte auftauchen können. So wurde das Stück „Norway.Today“ (Igor Bauersima) in der Spielzeit 2000/01 uraufgeführt. Danach tauchte es noch in 14 von 18 weiteren Spielzeiten auf, was einer Abdeckung von 77,8 % entspricht. Gefolgt wird „Norway.Today“ von „Das Fest“ (Thomas Vinterberg), welches in 11 von 18 möglichen Spielzeiten auftaucht (61,1 %), und „Ehrensache“ (Lutz Hübner), das in 7 von 13 möglichen Spielzeiten auftaucht (53,9 %). Bei Berechnung der Korrelation der Variablen Anzahl der Spielzeiten, in denen das neue Stück auftaucht und Anzahl der Spielzeiten, in denen das Stück hätte gespielt werden können, lässt sich ein Wert von r = 0,070 ermitteln, womit kein linearer Zusammenhang zwischen den beiden Variablen besteht. Diejenigen Theaterstücke, welche als Ur- oder Erstaufführung in der untersuchten Zeit gespielt wurden, werden somit kaum erneut gespielt. Es gelingt den NRW-Theatern nur sehr selten, neue Stücke im Repertoire zu etablieren.

Auch bei den Neuerungen stellt sich die Frage, inwieweit diese auf Vorlagen wie Büchern, Filmen oder anderen Theaterstücken beruhen. Betrachtet wurden hierfür die 50 am häufigsten gespielten Ur- oder Erstaufführungen (Tab. 2 und 3 dokumentieren die Top-20). Für diese Stücke wurde ermittelt, ob es sich um ein völlig neues Stück oder um die Verarbeitung einer vorhandenen Vorlage handelt. 13 der 50 untersuchten Stücke basieren auf einer Vorlage (26 %), wovon neun einen Roman und jeweils zwei einen Film oder ein anderes Theaterstück zur Vorlage hatten. Zur Illustration dieses Befundes werden die Spielhäufigkeiten der erfolgreichsten Neuerungen denjenigen Stücken gegenübergestellt, die im Zeitraum 1995/96–2017/18 allgemein am meisten aufgeführt wurden (Tab. 2 und 3). Deutlich wird, dass die „Kassenschlager“ alle auf klassische Theaterstücke zurückgehen, die zumeist in der Zeit vom 17. Jhd. bis Anfang des 20. Jhds. verfasst wurden. „Antigone“ von Sophokles geht sogar auf die Antike zurück. Solche Klassiker scheinen beim Publikum so beliebt, dass sie zum Teil jährlich auf den nordrhein-westfälischen Spielplänen stehen. Die höchsten Aufführungszahlen (892) erzielt „Kabale und Liebe“ von Friedrich Schiller (aus dem Jahr 1784). Demgegenüber ist „Norway.Today“ von Igor Bauersima (aus dem Jahr 2000) mit 369 Aufführungen die am häufigsten gespielte Neuerung.

**Table 2:** 

Erst- oder Uraufführung	Anzahl Aufführungen	Durchschnittliche Aufführungszahl pro Spielzeit	Besucher pro Aufführung
Norway.Today	369	26	121
Shakespeares sämtliche Werke	325	30	251
Das Fest	205	19	261
Die 39 Stufen	136	34	244
Für mich soll’s rote Rosen regnen	123	25	201
*Die Werkstatt der Schmetterlinge*	119	40	92
*Der Junge mit dem Längsten Schatten*	113	38	36
Kommt ein Mann zur Welt	108	27	169
Kugeln überm Broadway	107	27	532
Willkommen	106	27	233
Dornröschen oder wie man hundert Jahre spinnt	101	–	282
*Hinter verzauberten Fenstern*	93	47	358
*Igraine Ohnefurcht*	93	47	28
*Windsturmreiter*	92	15	74
Brassed off – Mit Pauken und Trompeten	91	18	323
Ehrensache	88	13	142
*Undine die kleine Meerjungfrau*	87	29	358
*Vom Jungen der in ein Buch fiel*	86	43	279
*Das Mond-Ei*	85	22	39
Männerhort	83	12	152

Quelle: DBV (1997–2019b): Werkstatistiken. In der dritten Spalte wurde das arithmetische Mittel gebildet, wenn die Stücke mehr als einmal gespielt wurden. Kinder‑/Jugendtheaterstücke stehen *kursiv*

**Table 3:** 

Alle Stücke	Anzahl Aufführungen	Durchschnittliche Aufführungszahl pro Spielzeit	Besucher pro Aufführung
Kabale und Liebe	892	47	361
*Die Schneekönigin*	885	68	455
*Pinocchio*	707	59	413
Ein Sommernachtstraum	688	31	405
*Ronja Räubertochter*	674	56	557
Romeo und Julia	647	34	381
Die Dreigroschenoper	643	34	497
Hamlet	601	29	378
*Der Zauberer von Oz*	590	49	492
Was ihr wollt	509	30	338
Nathan der Weise	504	28	361
Antigone	487	26	246
Woyzeck	479	25	303
*Die Bremer Stadtmusikanten*	453	32	414
*Jim Knopf und Lukas der Lokomotivführer*	446	45	517
Liebesperlen	439	26	498
Der zerbrochne Krug	421	26	353
*Der Räuber Hotzenplotz*	417	52	449
Tschick	411	69	170
Maria Stuart	406	29	400

Quelle: DBV (1997–2019b): Werkstatistiken. In der dritten Spalte wurde das arithmetische Mittel gebildet. Kinder‑/Jugendtheaterstücke stehen *kursiv*

Auch die durchschnittlichen Aufführungszahlen pro gespielter Spielzeit sowie die Besucher pro Aufführung verdeutlichen, dass die Top-20 Neuerungen (durchschnittlich 32 Aufführungen und 209 Besucher pro Spielzeit) weniger häufig als die Top-20 aller Stücke (durchschnittlich 40 Aufführungen und 399 Besucher pro Aufführung) gespielt werden und damit weniger hohe Besucherzahlen erreichen können. Die niedrigere durchschnittliche Besucherzahl der Neuerungen kommt zum einen dadurch zustande, dass die Stücke durchschnittlich weniger oft aufgeführt werden (minus 20 %) und zum anderen könnte ein weiterer Grund sein, dass neue Stücke eher auf Nebenbühnen aufgeführt werden im Sinne einer Risikokalkulation, falls das Stück nicht genügend Publikum anzieht.

Weiterhin ist auffällig, dass es sich bei zahlreichen der am häufigsten gespielten Stücke um Kinder- oder Jugendtheaterstücke handelt (Tab. 2 und 3, Titel kursiv). In der Gruppe aller Theaterstücke lassen sich sieben von den gelisteten Top-20 (35 %) dem Kinder‑/Jugendtheater zuordnen und in der Gruppe der E‑/U-Stücke acht (40 %). Dieser Befund ist für die Interpretation einer Innovationskrise bedeutsam, denn ein großer Teil der Top-20-Stücke adressiert Eltern mit Kindern sowie Jugendliche und erzielt damit eine Nachfrage, welche sich nicht nur in den hohen Aufführungszahlen, sondern auch in den vor allem in der Listung enthaltenen Top-20 aller Stücke (Tab. 3) hohen Besucherzahlen niederschlägt: Die sieben gelisteten Kinder‑/Jugendstücke erreichen insgesamt eine Besucherzahl von 1.965.578 und die restlichen Stücke 2.611.298, womit die durchschnittliche Besucherzahl von Kinder‑/Jugendstücken deutlich höher liegt (280.797 zu 200.869).

Hier schließt die Frage an, ob die Erneuerung des Theaterprogramms durch die Aufnahme von Erst- und Uraufführungen sowie die Wiederaufführung jener Neuerungen (= Innovationen) tatsächlich ausreichend ist, um neues Publikum zu gewinnen und der Nachfragekrise in Form des Besucherdefizits entgegenzuwirken, wenn es doch vor allem die konventionellen Klassiker sind, die durchschnittlich viele Besucher anlocken (Tab. 2 und 3). Diese Frage soll mithilfe von Tab. 4 beantwortet werden, welche auf Basis der Daten aus Tab. A3 im Online-Anhang berechnet wurde: Hierfür haben wir die Veränderung zwischen den beiden Zeiträumen 2005/06–2009/10 und 2013/14–2017/18 hinsichtlich der Anzahl an Besuchern insgesamt von Neuerungen (Ur- und Erstaufführungen) sowie Innovationen und vergleichend dazu die Publikumszahlen der restlichen Stücke (also exklusive der Neuerungen und Innovationen) betrachtet (Tab. 4). Die Innovationen sind in Anlehnung an die abhängige Variable 2 (AV2) folgendermaßen definiert: Wird eine Ur- oder Erstaufführung innerhalb der nachfolgenden zehn Jahre erneut gespielt, ist es eine Innovation. Wird eine Neuerung mehr als zehn Jahre nach ihrer Premiere nochmals gespielt, wird sie nicht mehr als Innovation gewertet, sondern als ein Stück, welches sich im Repertoire der nordrhein-westfälischen Theater etabliert hat. Hieraus ergibt sich ein beobachtbarer Zeitraum von 2005/06 bis 2017/18, da erst für die Spielzeit 2005/06 rückwirkend zehn Jahre (1995/96–2004/05) hinsichtlich der Ur- und Erstaufführungen analysiert werden können.

**Table 4:** 

	2005/06–2009/10	2013/14–2017/18	Absolute Veränderung	Prozentuale Veränderung (%)
Anzahl an Besucher von Neuerungen/Innovationen insgesamt	152.108	179.193	+27.084	+18
Anzahl an Besucher von Stücken (exkl. Neuerungen/Innovationen) insgesamt	1.249.072	1.102.134	−146.937	−12
Durchschnittliche Anzahl an Neuerungen/Innovationen pro Spielzeit	79	108	+29	+36
Durchschnittliche Anzahl an Neuerungen (exkl. Innovationen) pro Spielzeit	54	66	+12	+22
Durchschnittliche Anzahl an Stücken (exkl. Neuerungen/Innovationen) pro Spielzeit	430	363	−67	−12

Quelle: DBV (1997–2019b): Werkstatistiken

Folgendes Beispiel soll die Definition des hier genutzten Innovationsbegriffs illustrieren: Das Stück „Kochen mit Elvis“ von Lee Hall wurde in der Spielzeit 1999/00 im Schauspiel Essen als Erstaufführung gespielt, womit es in dieser Spielzeit eine Neuerung ist. In den nachfolgenden zehn Spielzeiten (2000/01–2009/10) wurde das Stück noch in drei weiteren Spielzeiten aufgeführt (2000/01, 2001/2002 und 2002/2003). 2017/18 wurde das Stück nochmals aufgeführt. Da dieser Zeitpunkt allerdings nicht in dem Zeitraum von zehn Jahren ab Premierenjahr liegt, handelt es sich in der Spielzeit 2017/18 auch nicht mehr um eine Innovation, sondern um ein Stück des Repertoires in NRW.

Tabelle 4 zeigt die auf diese Weise berechneten Gesamtzahlen, wobei wir unseren Fokus auf die relativen Zahlen legen: Im ersten Zeitraum 2005/06–2009/10 liegt die Anzahl an Besuchern von Neuerungen/Innovationen insgesamt bei 152.108 und für die übrigen Stücke (also exkl. Neuerungen und Innovationen) bei 1.249.072. Im Vergleichszeitraum 2013/14–2017/18 wächst die Anzahl der Besucher von Neuerungen/Innovationen um 18 % auf 179.193 (plus 27.084) an, während sich die Besucherzahl der restlichen Stücke um minus 12 % auf 1.102.134 (minus 146.937) verringert. Im Vergleich dazu ist die Anzahl der Neuerungen/Innovationen pro Spielzeit um 36 % von 79 auf 108 angestiegen. Gleichzeitig sind die restlichen Stücke um minus 12 % von 430 auf 363 gesunken.

Unterstrichen wird der schon in Abschn. 2 mithilfe der Strukturdaten aufgeworfene Befund, dass auf der einen Seite das Angebot (Anzahl Stücke) erhöht wurde, während die Nachfrage (Anzahl Besucher) kontinuierlich sinkt. Jedoch lässt sich dieser Befund nun differenzierter betrachten: Auf den ersten Blick wirkt es so, als gäbe es eine Überproduktion an Theaterstücken (Schmidt 2017). Jedoch kann mittels Tab. 4 gezeigt werden, dass Neuerungen und Innovationen neues Publikum erschließen, wenn auch das Angebot auf dieser Ebene ausgeweitet wird. Um eine Überproduktion zu vermeiden, müssten systematisch alte Stücke aus dem Repertoire entfernt und durch Neuerungen ersetzt werden. Eine Kürzung des Angebots würde darüber hinaus Ressourcen freisetzen (Haselbach et al. 2012).

Gerlach-March (2011) findet in dem Zusammenhang im Rahmen einer Fallstudie heraus, dass der britische Theatersektor hinsichtlich der Anzahl der Uraufführungen innovativer abschneidet als der deutsche Sektor. Ihre Deutung lautet, dass obwohl der deutsche Theatersektor stärker staatlich subventioniert wird, dies nicht automatisch in Neuerungen mündet. Es spielen also vor allem kulturpolitische Vorgaben und Traditionen eine Rolle. So ist beispielsweise das Autorentheater in Großbritannien weit verbreitet, bei welchem Autoren einen großen Einfluss auf die Spielplangestaltung nehmen, während in Deutschland vor allem das Regietheater vorzufinden ist, bei welchem Regisseure großen Einfluss auf die Inszenierungen nehmen. Möglicherweise führen kulturpolitische Vorgaben wie in Großbritannien dazu, dass Theater mehr Neuerungen in ihre Repertoires aufnehmen.

Zusammenfassend können die vorangegangenen deskriptiven Befunde als Beleg für eine Innovationskrise der staatlichen NRW-Theater interpretiert werden. Es gelingt den Theatern nur selten, neue Stücke zu Publikumserfolgen zu machen. Zwar werden einige Stücke (z. B. „Norway.Today“, „Das Fest“ oder „Shakespeares sämtliche Werke“) zu Publikumserfolgen, aber im Verhältnis zur Zahl aller Neuerungen schaffen es nur wenige Stücke ins Repertoire, obwohl Tab. 4 zeigt, dass eine Nachfrage nach neuen, innovativen Theaterstücken existiert. Zudem konnten wir zeigen, dass vor allem Jugendliche sowie Kinder und ihre Eltern eine Zielgruppe sind. Mandel (2021) bestätigt jene Nachfrage mithilfe einer Bevölkerungsumfrage sowohl unter Theater- als auch Nichttheatergängern hinsichtlich ihrer Erwartungen an die Theater. Die meistgenannte Erwartung (89 %) hinsichtlich der Spielplangestaltung war der Wunsch nach „Programmen für Kinder und Jugendliche“. Darüber hinaus gaben 66 % der Befragten an, dass sie sich „aktuelle Stücke und künstlerische Experimente“ wünschen. Zusätzlich fand Mandel (2020) heraus, dass es vor allem die 18- bis 39-Jährigen sind, die sich aktuelle und experimentelle Stücke wünschen. Möglicherweise könnten aufbauend auf dieser Erwartungshaltung mehr Theaterbesucher gewonnen werden, wenn erstens vor allem mehr Ur- und Erstaufführungen (im Sinne aktueller Stücke und künstlerischer Experimente) und zweitens mehr Kinder- und Jugendstücke auf die Bühnen gebracht würden.

### Erklärung von Neuerungen und Innovationen

Zur Hypothesenprüfung wurden Regressionen mittels STATA (Version 16) durchgeführt. Wir erläutern nachfolgend zunächst unsere Analysestrategie zur Untersuchung der AV1 und gehen anschließend näher auf die Ergebnisse anhand der zuvor aufgestellten *H 1* bis *H 4* ein. Danach erläutern wir unsere Analysestrategie zur AV2 und benennen die Ergebnisse für *H 5*.

Für die Analyse der AV1 haben wir eine OLS-Regression (Tab. 5), mehrere Robustheitstests und eine Fixed-Effects-Regression (FE) gerechnet. Die UVs unserer Hauptanalyse (Tab. 5) sind links beginnend in absteigender Reihenfolge ihres univariaten R^2^-Werts aufgeführt, nachdem in Modell (1) und (2) zunächst die Kontrollvariablen aufgeführt werden. Für die Modelle wurden die robusten Standardfehler geschätzt, da nach dem Breusch-Pagan-Test die Annahme der Homoskedastizität der Regressionsresiduen verletzt ist. Dies gilt auch für die nachfolgenden OLS-Robustheitsanalysen. Unsere Hauptmodelle (6) und (7)^2^ in Tab. 5 weisen einen R^2^-Wert von 0,435 bzw. 0,420 und sehr niedrige *p*-Werte auf, wodurch sie eine signifikante Erklärungsgüte bei einem Signifikanzniveau von α = 0,001 erhalten. In dem genannten Modell sind alle UVs signifikant: Anzahl der Aufführungen, Subventionen, Wettbewerbsdruck, Anzahl der Abonnements und Anzahl der Privattheater. Zudem sind auch die zwei Kontrollvariablen signifikant: Anzahl der gespielten Stücke und Anzahl der Spielstätten. Darüber hinaus haben wir überprüft, ob die Logarithmierung der Variablen der OLS-Analyse (Tab. 5) zu veränderten Ergebnissen führt, was jedoch nicht der Fall ist. Eine Variation der OLS (Tab. 5) besteht darin, für die AV1 anstelle der absoluten Anzahl von Neuerungen die Anteilswerte von Neuerungen an allen Stücken zu nutzen (Tab. A5 im Online-Anhang): Modell (6) und (7) erzielen hierbei geringere R^2^-Werte als in Tab. 5 und die Signifikanzen der einzelnen UVs bleiben mit Ausnahme der Abonnements erhalten.

**Table 5:** 

Variablen	(1)	(2)	(3)	(4)	(5)	(6)	(7)
Anzahl der Stücke	0,085*** (0,012)	0,074*** (0,012)	−0,038** (0,014)	−0,037** (0,013)	−0,121*** (0,019)	−0,126*** (0,019)	−0,141*** (0,019)
Spielstätte	–	0,23*** (0,043)	0,255*** (0,04)	0,129** (0,047)	0,165** (0,048)	0,161** (0,048)	0,283*** (0,043)
Aufführungen (in 100)	–	–	1,010*** (0,106)	0,858*** (0,107)	0,865*** (0,105)	0,888*** (0,107)	0,958*** (0,11)
Subventionen (in 1 Mio. €)	–	–	–	0,058*** (0,014)	0,058*** (0,013)	0,089*** (0,014)	–
Wettbewerb	–	–	–	–	3,392*** (0,593)	3,223*** (0,571)	3,703*** (0,549)
Abonnements (in 1000)	–	–	–	–	–	−0,025*** (0,004)	−0,015*** (0,003)
Privattheater	–	–	–	–	–	–	0,185*** (0,043)
*N*	454	454	454	453	453	435	435
Prop > F	0,000	0,000	0,000	0,000	0,000	0,000	0,000
(adj) R‑squared	0,111	0,171	0,326	0,361	0,410	0,435	0,42
RMSE	2,412	2,332	2,105	2,053	1,975	1,947	1,973

Standardfehler in Klammern

*p < 0,05, **p < 0,01, ***p < 0,001

Weiterhin sind mehrere Robustheitstests für die Ergebnisse der OLS-Regressionsanalyse (Tab. 5) Teil unserer Analysestrategie. Erstens wurden die Werte der AV1 und UVs durch dreijährige und fünfjährige gleitende Mittelwerte ersetzt (Tab. A6 und A7 im Online-Anhang). Diese Berechnung glättet die jährlichen Schwankungen, welche unter Umständen das Modell stören können. Die Ergebnisse blieben jedoch unverändert, was für die Robustheit der mit dem OLS-Modell ermittelten Ergebnisse spricht (Tab. 5). Zweitens wurde die OLS-Regression (Tab. 5) in zwei Variationen durchgeführt (Tab. A8 und A9 im Online-Anhang): Zum einen wurde Köln als einwohnerstärkste und zum anderen Dinslaken als einwohnerschwächste Stadt aus der Analyse ausgeschlossen, um zu überprüfen, inwiefern ihr Ausschluss zu einer Veränderung der Ergebnisse führt. Ihr Ausschluss führte jedoch zu keiner Veränderung des R^2^-Werts des Modells, ebenso wenig, wie sich die Signifikanzwerte der UVs verändern. Die Ergebnisse der Tab. 5 sind somit sehr robust.

Zusätzlich haben wir eine Fixed-Effects-Regression gerechnet, in welcher die robusten Standardfehler (Driscoll-Kraay) geschätzt wurden (Tab. A10 im Online-Anhang). Für die FE sprach der Hausman-Spezifikationstest, welcher eine Random-Effects-Regression ablehnte. Mittels der FE haben wir jahres- und theaterspezifische Effekte überprüft. Die Signifikanzwerte der Variablen aus Modell (6) und (7) verschlechtern sich bei den theatergebundenen Variablen im FE-Modell gegenüber dem OLS-Modell leicht, im Wesentlichen wird das OLS-Modell mit Ausnahme der Privattheater bestätigt. Weiter wurden auf Ebene der kategorialen Variablen Spielzeiten (= 23 Jahre) und Theater (*n* = 20) Dummy-Variablen erzeugt. Die jahres- und theaterspezifischen Effekte der Dummy-Koeffizienten, welche mittels entsprechender Regressionen berechnet wurden, zeigen keinen nennenswerten Einfluss auf die Modelle. Dies spricht zusätzlich für die Robustheit der OLS, da die Einflüsse über die Theater und Spielzeiten hinweg gleichbleiben. Im Folgenden werden die Ergebnisse der Regressionsanalysen mithilfe der Hauptmodelle (6) und (7) (Tab. 5) auf die Hypothesen bezogen.

*H 1:* In Modell (6) übt die Variable Aufführungen einen positiven Einfluss (*p* < 0,001) auf die AV1 aus. Der Koeffizient in Modell (6) beträgt 0,888 und die UV ist auf 100 skaliert, was bedeutet, dass 113 zusätzliche Aufführungen mit einer zusätzlichen Neuerung einhergehen.^3^ Je höher die Anzahl der Aufführungen, umso höher ist die Möglichkeit (oder der Bedarf) für neue Stücke. Zugleich übt die Kontrollvariable Stücke einen signifikant negativen Einfluss (*p* < 0,001) auf die AV1 Erst‑/Uraufführungen aus. Der Koeffizient beträgt −0,126, was bedeutet, dass wenn acht bereits bekannte Stücke dem Spielplan hinzugefügt werden, ein neues Stück weniger inszeniert wird. Je mehr Stücke gespielt werden, umso mehr neue Stücke werden aus dem Spielplan gedrängt. Theater, die wenige unterschiedliche Stücke auf den Spielplan setzen und diese besonders häufig aufführen, sind somit besonders offen für Neues.

Zwei Beispiele mögen diesen Sachverhalt verdeutlichen. Das Düsseldorfer Schauspielhaus führte über den Analysezeitraum (1995/96–2017/18) insgesamt 923 verschiedene Stücke auf, davon 140 Ur- und Erstaufführungen. Obwohl die Variable Stücke in der OLS einen negativen Einfluss hat und in dem genannten Beispiel quantitativ hoch ausfällt, wird ihr Einfluss im Falle von Düsseldorf durch die hohe Anzahl an Aufführungen (*N* = 15.682) kompensiert. Im Kontrast dazu weist das Schauspielhaus in Bochum deutlich mehr Stücke als Düsseldorf auf (*N* = 1023), aber gleichzeitig weniger Aufführungen (*N* = 11.322). Düsseldorf hat daher einen höheren Rangplatz als Bochum (Tab. A2).

*H 3:* Subventionen haben einen positiven Koeffizienten (*p* < 0,001). Der Koeffizient von 0,089 besagt, dass rechnerisch 11,2 Mio. € notwendig wären, damit ein Theater ein weiteres neues Stück in den Spielplan aufnimmt. Damit wird *H 3* zwar bestätigt, allerdings ist die Effektstärke dieser UV sehr gering. Eine Erhöhung von Neuerungen mithilfe zusätzlicher Subventionen wäre nur unter Inkaufnahme sehr hoher Kosten möglich. Zugleich weist die Kontrollvariable Spielstätte einen positiven Koeffizienten (*p* < 0,001) auf. Der Koeffizient von 0,161 besagt, dass sechs zusätzliche Spielstätten notwendig wären, um ein neues Theaterstück auf den Spielplan zu setzen.

In diesem Zusammenhang ist noch einmal der deskriptive Befund zu nennen, dass seit Mitte der 2000er-Jahre die Anzahl der Spielstätten der NRW-Theater und die Zahl der Neuerungen substanziell ansteigt. Die Regressionsanalyse bestätigt somit den deskriptiven Befund. Allerdings ist die Effektstärke dieser UV relativ gering. Eine Erhöhung von Neuerungen mithilfe neuer Spielstätten ist (aus Kostengründen) nur in einem eher begrenzten Umfang möglich. Theater, welche viele Spielstätten betreiben und in hohem Maße subventioniert werden, erscheinen somit grundsätzlich offener für Neues als Theater mit nur einer Spielstätte und wenig staatlicher Unterstützung. Allerdings sollte man hier die geringen Effektstärken berücksichtigen. Im FE-Modell (Tab. A10) sind diese beiden UVs auch nur schwach signifikant. Ihre Bedeutung ist somit eher zu relativieren. Auch kulturpolitisch wäre es aufgrund der hohen Kosten keine vielversprechende Idee, die Produktion neuer Stücke bloß mit zusätzlichen Spielstätten und Subventionen zu fördern.

*H 4*^444^*:* Eine deutlich effektivere Möglichkeit, neue Stücke zu generieren, ergibt sich durch die UV Wettbewerb, die einen signifikant positiven Einfluss (*p* < 0,001) auf AV1 ausübt. Unseren Ergebnissen zufolge versuchen Theater, welche einem erhöhten Wettbewerbsdruck ausgesetzt sind, sich von der Konkurrenz durch Neuerungen im Programm abzuheben. Den höchsten durchschnittlichen CPI-Wert (1995–2018) erreichen das Bochumer Schauspielhaus (1,04), das Düsseldorfer Schauspielhaus (0,83) und das Schauspiel Essen (0,70). Diese drei Theater sind somit dem stärksten Wettbewerbsdruck ausgesetzt. Sie liegen zentral in NRW und weisen eine hohe räumliche Nähe zueinander sowie auch zu anderen Theatern auf. Am wenigsten Wettbewerb sind dagegen die Bielefelder Bühnen (0,07), das Landestheater Detmold (0,06) und das Aachener Grenzlandtheater (0,04) ausgesetzt, was sich mit ihrer Randlage in NRW erklären lässt. Betrachtet man in dem Zusammenhang die Anzahl der Neuerungen (Tab. A2), wird deutlich, dass vor allem Düsseldorf (Rang 1), Bochum (Rang 5) und Essen (Rang 8) nicht nur einem erhöhten Wettbewerb ausgesetzt sind, sondern auch besonders viele Neuerungen verzeichnen können, während Detmold (Rang 16) und das Aachener Grenzlandtheater (Rang 18) deutlich weniger Neuerungen aufweisen. Damit widerlegen wir *H 4*, welche von Jensen und Kim (2014) abgeleitet wurde. Jedoch sei an dieser Stelle nochmals anzumerken, dass die Autoren in ihrer Studie Konventionalität (und nicht Neuerungen wie im vorliegenden Beitrag) untersuchen.

Ebenfalls signifikant ist die Variable Privattheater bei einem Koeffizienten von 0,185 (Modell 7), was bedeutet, dass fünf zusätzliche Privattheater in derselben Stadt mit einer zusätzlichen Neuerung einhergehen. Dies ist ein plausibler Wert, denn die Variable nimmt in unserem Datensatz einen maximalen Wert von 23 (Köln) an. Dennoch sei an dieser Stelle nochmals zu erwähnen, dass die Übermittlung der Daten für Privattheater auf freiwilliger Basis geschieht, weshalb erhebliche Datenlücken und eine Selbstselektion bei den Daten vorliegen können.

*H 2:* Die Variable Abonnements ist ebenfalls hochsignifikant (*p* < 0,001) und der Koeffizient fällt mit einem Wert von −0,025 je 1000 Abonnements negativ aus (Modell 6). Das bedeutet, dass 40.000 Abonnements pro Theater benötigt würden, um eine Neuerung zu erhalten. Allerdings ist dies sehr unrealistisch: Die durchschnittliche Abonnementszahl der Theater in NRW liegt bei 32.000 (max. 87.000). Daher gilt auch hier, wie bereits bei *H 3*, dass *H 2* zwar bestätigt wird, die geringe Effektstärke aber die Absenkung von Abonnements nicht als sinnvolle Einflussgröße für die Produktion neuer Stücke erscheinen lässt.

In einem weiteren Schritt wurde die AV2 (Innovation) mithilfe einer Poisson-Regression untersucht (Tab. 6). Hierbei fällt *N* = 254 um knapp 200 Einheiten kleiner aus als bei der vorangegangenen OLS: Um nämlich zu ermitteln, ob eine Ur- oder Erstaufführung in den zehn Folgejahren in mindestens einem weiteren der untersuchten NRW-Theater aufgeführt wurde, konnten nur die Ur- oder Erstaufführungen der Spielzeiten 1995/96–2007/08 berücksichtigt werden, da nur für diese Spielzeiten zehn weitere Beobachtungsjahre zur Verfügung stehen. Dies bedeutet, dass wir für die Analyse der AV2 nur einen Ausschnitt des Gesamtdatensatzes betrachten (1995/96–2007/08). Die maximale Ausprägung der AV2 beträgt *n* = 2. Ein Theater führt also maximal zwei Neuerungen pro Spielzeit auf, welche in den nachfolgenden zehn Spielzeiten von mindestens einem anderen NRW-Theater übernommen wurden.

**Table 6:** 

Variablen	(1)	(2)
Stücke pro Jahr	−0,007* (0,003)	−0,006 (0,003)
Anzahl Neuheiten (AV1)	–	0,343*** (0,05)
*N*	260	254
Prop > Chi^2^	0,02	0,000
LR Chi^2^	5,41	47,70
Pseudo R^2^	0,018	0,161

Standardfehler in Klammern

*p < 0,05, **p < 0,01, ***p < 0,001

Für die Durchführung einer Poisson-Regression spricht der Pearson Goodness-of-Fit-Test, welcher auf einen soliden „model-fit“ hindeutet und von der Annahme abhängt, dass die bedingte Varianz und der Mittelwert der Beobachtungen gleich sind. Wie schon bei der OLS-Regression wurden auch hier zur Überprüfung der Robustheit Vergleichsanalysen mit gleitenden Mittelwerten gerechnet, die die Ergebnisse der nichtgeglätteten Daten weitgehend bestätigten (Tab. A12 und A13 im Online-Anhang). Um eine Überdispersion zu vermeiden und zur Überprüfung der Robustheit wurde eine negative Binomial-Regression durchgeführt, deren Ergebnisse kaum von denen in Tab. 6 abweichen, was ebenfalls für die Robustheit der Ergebnisse in Tab. 6 spricht (Tab. A14 im Online-Anhang).

*H 5:* Wie Modell (2) zu entnehmen ist, hat die bisherige AV1 in der Poisson-Regression einen positiven Einfluss auf die AV2 (*p* < 0,001). Dies bedeutet, dass mit steigender Zahl von Neuerungen in allen NRW-Theatern (Organisationsfeld) die Chance für eine konkrete Ur- oder Erstaufführung eines konkreten Theaters steigt, mittel- und langfristig ins Repertoire aufgenommen zu werden. Ein für Neuerungen offenes Organisationsfeld ist bei der Durchsetzung neuer Stücke somit wichtig. Das bedeutet zugleich aber auch, dass einzelne Theater nur wenig alleine ausrichten können, wenn andere Theater nicht auch Neuerungen auf ihren Spielplan setzen. Insoweit würde es nicht reichen, wenn nur wenige „Exzellenztheater“ viele Neuerungen produzierten, sondern diese sollten in der Breite des Theaterfeldes als kulturelles Muster vorhanden sein. Erst dann sind Innovationen möglich.

## Diskussion

Das Ziel unserer Studie war es zu untersuchen, welche Faktoren die Repertoireentscheidungen hinsichtlich Ur- und Erstaufführungen beeinflussen. Die kultursoziologische Forschung hat die Innovationstätigkeit des staatlichen Theatersektors in Deutschland im Gegensatz zum angelsächsischen Raum bisher kaum untersucht. Daher greift die Studie vor allem internationale Arbeiten und die darin untersuchten Variablen und Hypothesen auf, um unsere Ergebnisse für NRW anschlussfähig zu machen. Der vorliegende Aufsatz versucht die vorrangig für Deutschland bestehende Forschungslücke am Beispiel von NRW teilweise zu schließen und ergänzt die bekannten Befunde zur Alterung des Publikums und rückläufigen Besucherzahl (Nachfrageseite) um Befunde zur Angebotsseite.

Von den rund 12 % Ur- und Erstaufführungen, die im Zeitraum 1995/96–2007/08 auf den staatlichen Bühnen NRWs aufgeführt wurden, schaffen es in den nachfolgenden Jahren nur sehr wenige ins Repertoire (anderer NRW-Theater). Es sind aber vor allem Neuerungen und Innovationen, die neue Besucherschichten erschließen. So konnten wir zeigen, dass unter denjenigen neuen Stücken, die hohe Aufführungszahlen erzielen, sich viele Stücke finden, welche auf Kinder- oder Jugendbüchern basieren, was auf Eltern mit Kindern als Zielgruppe hindeutet. Hierbei sollte jedoch bedacht werden, dass ein Theaterstück zwar ein Kinder- oder Jugendbuch zur Vorlage haben kann, die Inszenierung des Stücks aber auch generell an Erwachsene gerichtet sein kann. Um diesbezüglich zu einer empirisch fundierten Aussage zu gelangen, könnten in zukünftigen Studien Spielplandaten anhand qualitativer Merkmale oder der Uhrzeit (Kinder-Vorstellungen finden tendenziell vor 18 Uhr statt) überprüft werden.

Unsere deskriptiven Befunde legen nahe, dass sich seit Mitte der 1990er-Jahre in NRW ein deutlicher Rückgang der Nachfrage (Zuschauer) in staatlichen Theatern erkennen lässt. Auf diese Nachfragekrise haben die NRW-Theater mit einer erheblichen Ausweitung ihres Angebots reagiert. In dem untersuchten Zeitraum wurden erstens deutlich mehr Spielstätten betrieben (plus 41 %), zweitens zahlreiche neue Stücke (plus 38 %) und drittens mehr Stücke insgesamt (plus 26 %) ins Programm übernommen. Unserer Einschätzung nach haben die Theater versucht, auf diese Weise dem Publikumsschwund entgegenzuwirken. Allerdings konnten wir zeigen, dass die meisten der neuen Stücke sich nicht im Repertoire etabliert haben. Wenn man somit von einer „Theaterkrise“ spricht, dann handelt es sich nicht nur um ein sozialstrukturelles Phänomen. Vielmehr liegt der Theaterkrise auch ein Angebotsproblem zugrunde: Die Dominanz bekannter Stücke und ein zu geringes Maß an Neuerungen und Innovationen. Hierfür spricht der Befund, dass die Besucherzahl der Neuerungen und Innovationen insgesamt über den beobachteten Zeitraum anwächst (plus 18 %), während die Besucherzahl der restlichen Stücke sinkt (minus 12 %). Ein mögliches Anschlussprojekt wäre die Untersuchung, welche Besucher durch Neuerungen und Innovationen gewonnen werden bzw. sich für diese Art von Theaterstücken besonders interessiert zeigen und somit für den genannten Besucherzuwachs verantwortlich sind.

Die von uns durchgeführten Regressionsanalysen legen darüber hinaus den Schluss nahe, dass die Erneuerungsfähigkeit kommunaler oder landeseigener Theater am Beispiel von NRW erhöht werden könnte, wenn sowohl einzelne Theater als auch der ganze staatliche Theatersektor deutlich mehr neue Stücke ins Programm hineinnehmen und zugleich alte Stücke absetzen würden. Die Erneuerungsquote von rund 12 % neuer Stücke (Ur- und Erstaufführungen) ist offensichtlich zu niedrig. Etwas zugespitzt könnte man sagen, dass die Theaterprogramme in NRW mit „alten Stücken“ ausgelastet sind und zu wenig Platz für Neues haben. Die Ausweitung der Spielstätten und die erheblichen Anstrengungen der Theater seit Mitte der 2000er-Jahre, neue Stücke auf die Bühne zu bringen, gingen bislang nicht zulasten der „alten Stücke“. Es hat also keine Verdrängung stattgefunden, die notwendig wäre, um den Spielraum für neue Inszenierungen und Stücke substanziell zu öffnen, und zwar sowohl auf der Ebene konkreter Theater als auch auf der Ebene des gesamten Organisationsfeldes. Je mehr bislang gebundene Kapazitäten freigesetzt würden, umso mehr könnte jedes Theater nicht nur selbst viel mehr Neuerungen ins Programm hineinnehmen, sondern zugleich auch anderswo gespielte Neuerungen aufgreifen und damit zu einem festen Bestandteil des Repertoires machen.

Unsere Analysen haben auch gezeigt, dass eine bloße Erhöhung der Spielstättenanzahl oder der Subventionen keine Lösung für die Innovationskrise wäre. Die Koeffizienten unserer Regressionsanalysen deuten auf Größenordnungen hin, die kulturpolitisch nicht oder nur schwer umsetzbar wären (z. B. rechnerisch 11,2 Mio. € pro Ur- oder Erstaufführung). Demgegenüber zeigt Wettbewerbsdruck einen positiven Effekt auf die Anzahl von Neuerungen. Im dezentralen Wettbewerb befindliche Theater spielen neuartige Stücke, um sich von der Konkurrenz abzuheben. Die hohe Effektstärke der Wettbewerbsvariable legt auch nahe, dass es keine sinnvolle kulturpolitische Strategie wäre, mit zunehmendem Publikumsschwund einfach Theater zu schließen. Denn solche Schließungen hätten eine Verringerung des Wettbewerbs und damit auch der Neuerungsrate zur Folge.

Unsere Studie ergibt einige Befunde für den Theatersektor in NRW. Allerdings ist offen, inwieweit sich diese Ergebnisse für den gesamten deutschen Theatersektor (und über einen längeren Zeitraum) verallgemeinern lassen. NRW könnte insoweit ein besonderer Fall sein, als dass im Gegensatz zu den anderen deutschen Bundesländern eine vergleichsweise hohe Dichte von Theatern in direkter räumlicher Nähe vorherrscht. Daher wäre im Rahmen einer Vollerhebung zu überprüfen, inwiefern die bisherigen Befunde regional differenziert werden müssen. Auch ließe sich durch eine Ausweitung auf ganz Deutschland die Übernahme von Neuerungen ins Repertoire aller staatlichen Theater und damit die Innovationsvariable noch breiter erfassen.

Mit unserem Datensatz können wir nur Innovationen innerhalb von NRW betrachten, weshalb Fälle, in denen ein NRW-Theater eine Neuerung eines Theaters aus einem anderen Bundesland übernimmt und umgekehrt, unberücksichtigt blieben. Hierdurch entsteht möglicherweise eine Verzerrung, die durch eine Vollerhebung aller Theater in Deutschland vermieden werden könnte. Darüber hinaus sollte an dieser Stelle festgehalten werden, dass in unserer Analyse staatliche Theater untersucht wurden. Da die Daten der Privattheater auf optionalen Selbstauskünften basieren und daher nur unvollständig vom Deutschen Bühnenverein erfasst werden, könnte ein mögliches – und dann aber längerfristig angelegtes – Forschungsvorhaben darin bestehen, durch weitere Recherchen in Theater- und Stadtarchiven eine deutlich verbesserte Datenbasis zu Privattheatern zu schaffen.

Eine weitere Forschungslücke besteht darin zu prüfen, ob andere deutsche Kulturbereiche, wie beispielsweise Opernhäuser und Sinfonieorchester, ebenfalls von der hier diagnostizierten Innovationskrise betroffen sind. Ein weiterer möglicher Forschungsschritt könnte in einer vergleichenden Analyse der Neuerungen mit jener Konformität im Sinne von DiMaggio und Stenberg (1985a, b) bestehen.

Abschließend sei noch anzumerken, dass unsere Analyse die Situation vor der Covid-19-Pandemie beschreibt. Die durch die Schließung aller Kultureinrichtungen („Lockdown“) verursachte Zwangspause wird ein Wiederhochfahren der staatlichen Kulturbetriebe vermutlich vor gewisse Probleme stellen. Hierzu zählt einerseits, dass aufgrund der hohen zusätzlichen Verschuldung der öffentlichen (kommunalen) Haushalte eine Prioritätensetzung zulasten des Kultursektors nicht unwahrscheinlich erscheint und die Frage nach der Legitimität dieser Kultursubventionen für ein sozialstrukturell hochselektives Publikum zur Diskussion kommen könnte. Die in der Einleitung erwähnte Finanzkrise des Theatersektors könnte sich daher noch verschärfen: So schreiben Alexander und Spiegel (2021), dass aufgrund von Etatkürzungen, bedingt durch die Pandemie, in vielen Städten Verunsicherung herrscht, wie teure Theaterbauprojekte finanziert werden sollen. Weiter stellen sie fest, dass es für ein Theater von existenzieller Bedeutung ist, den Spielbetrieb konstant aufrechtzuerhalten, da einmal verlorene Abonnenten nur schwer zurückzugewinnen sind.

Hinzu kommt, dass sich das bisherige (im Schwinden begriffene) Publikum während der Lockdowns an neue kulturbezogene Onlineformate gewöhnt hat und nur teilweise in die Theater (und generell: Kulturstätten) zurückkehren könnte. Denn die im Publikum überproportional repräsentierten älteren Geburtskohorten sind gesundheitlich besonders von der Pandemie gefährdet gewesen. Möglicherweise erzeugt somit die Zwangspause einen Druck zur Neuausrichtung des Programms. Auf diese Weise könnten neue, insbesondere jüngere Besucherschichten erschlossen werden. Die sozialwissenschaftliche Forschung sollte den pandemiebedingten Strukturwandel des Theatersektors durch weitere empirisch-analytische Studien begleiten und auf diese Weise an der Erstellung einer Grundlage für rationale kulturpolitische Entscheidungen mitwirken.

## Supplementary Information





## Footnotes

1 Im Folgenden wird das generische Maskulinum verwendet. Es ist immer sowohl die männliche als auch die weibliche Form gemeint.

2 So zeigte sich bei der Durchsicht der Korrelationen (Tab. A4 im Online-Anhang), dass die Variablen Subventionen und Privattheater korreliert sind (r = 0,703), weshalb diese in Modell (6) und (7) getrennt analysiert werden.

3 Der Wert von 113 ergibt sich wie folgt: 1 / 0,888 = 1,126. Da die Aufführungen in 100 angegeben sind, kommt man durch Aufrunden des Ergebnisses auf 113 Aufführungen. Zu beachten ist hierbei, dass es sich um einen Durchschnittswert handelt. Dies bedeutet nicht, dass zwingend 113 Aufführungen nötig sind, um die abhängige Variable um eine Einheit (eine Neuerung) zu erhöhen, sondern dass der errechnete Wert für die unterschiedlichen Städte oder Theater variieren kann (da es sich um einen Durchschnittswert handelt).

444 Wir haben den CPI unserer OLS-Regression (Tab. 5) zusätzlich auf Basis von Kilometern anstatt Meilen erneut berechnet (Tab. A11 im Online-Anhang). Die Ergebnisse beider Tabellen stimmen im Wesentlichen überein, außer dass der Koeffizient der Variable Wettbewerb größer ausfällt, wenn er auf der Basis von Kilometern berechnet wird. Um die Vergleichbarkeit zur bestehenden Literatur, welche allesamt den Wettbewerb anhand von Meilen berechnet, zu gewährleisten, berechnen wir in unserer Haupt-OLS ebenfalls den CPI auf der Basis von Meilen.
