# Supplementary material for: Innovation Crisis in Public Theatre? A Longitudinal Study of Theaters in North Rhine-Westphalia, 1995–2018
Source: Kolner Z Soz Sozpsychol. 2022 Jul 27;74(2):203–32. [Article in German] doi: 10.1007/s11577-022-00846-3 (PMC9326436; doi:10.1007/s11577-022-00846-3)
Supplement: Supplementary file 1 [file 11577_2022_846_MOESM1_ESM.docx]

**Anhangtabellen**

**Tabelle A1: Kennzahlen zu kommunalen und landeseigenen Theatern in Deutschland**

|  | 1995/ 1996 | 2000/ 2001 | 2005/ 2006 | 2010/ 2011 | 2015/ 2016 | 2017/ 2018 |
| --- | --- | --- | --- | --- | --- | --- |
| Anzahl Theaterunternehmen | 154 | 150 | 143 | 140 | 143 | 143 |
| Anzahl Spielstätten | 655 | 728 | 793 | 890 | 815 | 838 |
| Anzahl Sitzplätze in Tsd. | 242 | 260 | 278 | 278 | 258 | 266 |
| Eigene Veranstaltungen (Standort) | 61.914 | 62.989 | 62.749 | 67.755 | 67.257 | 65.794 |
| Vollpreis-/Tageskarten in Mio. | 6.720 | 7.321 | 6.813 | 7.968 | 8.337 | 8.097 |
| Abonnements in Tsd. | 4.727 | 4.392 | 3.885 | 3.423 | 3.205 | 3.131 |
| Besucher*innen am Standort in Tsd. | 20.550 | 20.057 | 18.769 | 19.017 | 19.078 | 18.529 |
| Reale Betriebseinnahmen | 315.131 | 344.740 | 353.454 | 376.889 | 386.665 | 378.589 |
| Zuschüsse priv. Einrichtungen | 7.615 | 11.462 | 16.307 | 23.769 | 26.858 | 27.080 |
| Zuschüsse (Bund und Länder) | 1.988.545 | 1.925.869 | 1.827.501 | 1.836.607 | 1.908.026 | 1.940.139 |
| Einnahmen insgesamt | 2.328.403 | 2.294.059 | 2.214.431 | 2.254.708 | 2.344.159 | 2.303.723 |

*Quelle: DBV (1997-2019); Finanzangaben in Tsd. Euro und inflationsbereinigt (Basis: 1995)*

**Tabelle A2: Anzahl und Anteil von Neuerungen in NRW-Theater, 1995-2018**

| Theater | Anzahl Neuerungen | Anteil Neuerungen an allen Stücken | Rang Anzahl | Rang Anteil |
| --- | --- | --- | --- | --- |
| Düsseldorfer Schauspielhaus | 140 | 0,152 | 1 | 5 |
| Köln Bühnen der Stadt | 128 | 0,201 | 2 | 1 |
| Dortmund Theater | 125 | 0,177 | 3 | 3 |
| Bonn Theater der Bundesstadt | 90 | 0,155 | 4 | 4 |
| Bochum Schauspielhaus | 90 | 0,088 | 5 | 16 |
| Bielefeld Bühne und Orchester der Stadt | 85 | 0,180 | 6 | 2 |
| Oberhausen Theater | 80 | 0,135 | 7 | 7 |
| Essen Schauspiel | 65 | 0,109 | 8 | 13 |
| Münster Städtische Bühne | 58 | 0,120 | 9 | 10 |
| Aachen Stadttheater | 53 | 0,127 | 10 | 8 |
| Neuss Rheinisches Landestheater | 35 | 0,092 | 11 | 15 |
| Krefeld und Mönchengladbach Theater | 32 | 0,079 | 12 | 18 |
| Castrop-Rauxel Westfälisches Landestheater | 30 | 0,057 | 13 | 19 |
| Wuppertaler Bühnen | 29 | 0,088 | 14 | 17 |
| Hagen Theater | 28 | 0,111 | 15 | 12 |
| Detmold Landestheater | 25 | 0,047 | 16 | 20 |
| Moers Schlosstheater | 25 | 0,102 | 17 | 14 |
| Aachen Grenzlandtheater | 21 | 0,117 | 18 | 11 |
| Duisburg Theater | 20 | 0,126 | 19 | 9 |
| Dinslaken Landestheater Burghofbühne | 18 | 0,045 | 20 | 21 |
| Mülheim an der Ruhr Theater an der Ruhr | 11 | 0,025 | 21 | 22 |
| Gelsenkirchen Musiktheater im Revier | 7 | 0,137 | 22 | 6 |

*Quelle: DBV (1997-2019b): Werkstatistiken.*

**Tabelle A3: Veränderungen von Angebot und Nachfrage pro Spielzeit**

| Spielzeit | Besucher*innen insgesamt von Neuerungen und Innovationen | Besucher*innen insgesamt exklusive Neuerungen und Innovationen | Besucher*innen insgesamt | Anzahl Neuheiten und Innovationen | Anzahl Stücke exklusive Neuheiten und Innovationen | Anzahl Stücke insgesamt |
| --- | --- | --- | --- | --- | --- | --- |
| 1995/1996 | 113.596 | 1.411.247 | 1.524.843 | 42 | 319 | 361 |
| 1996/1997 | 189.197 | 1.410.212 | 1.599.409 | 50 | 310 | 360 |
| 1997/1998 | 117.903 | 1.421.726 | 1.539.629 | 55 | 341 | 396 |
| 1998/1999 | 116.415 | 1.427.689 | 1.544.104 | 53 | 335 | 388 |
| 1999/2000 | 137.192 | 1.274.482 | 1.411.674 | 61 | 335 | 396 |
| 2000/2001 | 179.516 | 1.224.306 | 1.403.822 | 66 | 298 | 364 |
| 2001/2002 | 234.985 | 1.302.162 | 1.537.147 | 75 | 322 | 397 |
| 2002/2003 | 184.940 | 1.387.371 | 1.572.311 | 70 | 316 | 386 |
| 2003/2004 | 144.062 | 1.259.891 | 1.403.953 | 64 | 342 | 406 |
| 2004/2005 | 108.462 | 1.179.240 | 1.287.702 | 61 | 334 | 395 |
| 2005/2006 | 116.628 | 1.215.698 | 1.332.326 | 61 | 412 | 473 |
| 2006/2007 | 127.934 | 1.255.683 | 1.383.617 | 62 | 423 | 485 |
| 2007/2008 | 140.664 | 1.321.316 | 1.461.980 | 74 | 463 | 537 |
| 2008/2009 | 191.184 | 1.208.931 | 1.400.115 | 89 | 432 | 524 |
| 2009/2010 | 184.131 | 1.243.732 | 1.427.863 | 111 | 418 | 530 |
| 2010/2011 | 202.097 | 1.176.860 | 1.378.957 | 108 | 400 | 508 |
| 2011/2012 | 194.284 | 1.115.956 | 1.310.240 | 113 | 384 | 498 |
| 2012/2013 | 179.590 | 1.083.464 | 1.263.054 | 118 | 415 | 538 |
| 2013/2014 | 201.528 | 1.019.488 | 1.221.016 | 106 | 362 | 472 |
| 2014/2015 | 151.720 | 1.225.303 | 1.377.023 | 94 | 380 | 482 |
| 2015/2016 | 119.806 | 1.222.275 | 1.342.081 | 107 | 399 | 514 |
| 2016/2017 | 221.435 | 1.044.669 | 1.266.104 | 123 | 334 | 463 |
| 2017/2018 | 201.476 | 998.939 | 1.200.415 | 110 | 339 | 454 |

**Tabelle A4: Korrelationsanalyse**

|  | Neuer-ungen | Aufführ-ungen | Subven-tionen | Wettbe-werb | Spiel-stätte | Abon-nements | Stücke | Privat-theater |
| --- | --- | --- | --- | --- | --- | --- | --- | --- |
| Neuerungen | 1,000 |  |  |  |  |  |  |  |
| Aufführungen | 0,499* | 1,000 |  |  |  |  |  |  |
| Subventionen | 0,472* | 0,384* | 1,000 |  |  |  |  |  |
| Wettbewerb | 0,370* | 0,606* | 0,219* | 1,000 |  |  |  |  |
| Spielstätte | 0,298* | 0,084 | 0,569* | 0,042 | 1,000 |  |  |  |
| Abonnements | 0,049 | 0,125* | 0,524* | 0,009 | 0,252* | 1,000 |  |  |
| Stücke | 0,334* | 0,742 | 0,335* | 0,817* | 0,175* | 0,075 | 1,000 |  |
| Privattheater | 0,389* | 0,359* | 0,703* | 0,171* | 0,261* | 0,309 | 0,312* | 1,000 |

*Anmerkung*: *p < 0,05

**Tabelle A5: OLS-Regression: Anteil der Ur- oder Erstaufführungen an dem Stücken insgesamt (AV) mit geschätzten robusten Standardfehlern**

| **Variablen** | **(1)** | **(2)** | **(3)** | **(4)** | **(5)** | **(6)** | **(7)** |
| --- | --- | --- | --- | --- | --- | --- | --- |
| Anzahl der Stücke | -0,002** (0,001) | -0,002*** (0,001) | -0,005*** (0,001) | -0,006*** (0,001) | -0,009*** (0,001) | -0,01*** (0,001) | -0,01*** (0,001) |
| Spielstätte | - | 0,01*** (0,002) | 0,011*** (0,002) | 0,006* (0,003) | 0,008** (0,003) | 0,007** (0,003) | 0,011*** (0,002) |
| Aufführungen (in 100) | - | - | 0,032*** (0,004) | 0,027*** (0,004) | 0,027*** (0,004) | 0,028*** (0,001) | 0,029*** (0,004) |
| Subventionen (in 1 Mio. €) | - | - | - | 0,002** (0,001) | 0,002*** (0,006) | 0,003*** (0,001) | - |
| Wettbewerb | - | - | - | - | 0,148*** (0,024) | 0,144*** (0,024) | 0,161*** (0,023) |
| Abonnements (in 1.000) | - | - | - | - | - | -0,001* (0,000) | -0,001 (0,000) |
| Privattheater | - | - | - | - | - | - | 0,007*** (0,002) |
| N | 454 | 454 | 454 | 453 | 453 | 435 | 435 |
| Prop > F | 0,001 | 0,000 | 0,000 | 0,000 | 0,000 | 0,000 | 0,000 |
| (adj) R-squared | 0,027 | 0,089 | 0,179 | 0,206 | 0,259 | 0,261 | 0,263 |
| RMSE | 0,106 | 0,102 | 0,097 | 0,096 | 0,093 | 0,093 | 0,093 |

*Anmerkung*: *p < 0,05, **p < 0,01, ***p < 0,001. Standardfehler in Klammern.

**Tabelle A6: OLS-Regression AV1 mit dreijährig gleitenden Mittelwerten** **mit geschätzten robusten Standardfehlern**

| **Variablen** | **(1)** | **(2)** | **(3)** | **(4)** | **(5)** | **(6)** | **(7)** |
| --- | --- | --- | --- | --- | --- | --- | --- |
| Anzahl der Stücke | 0,115*** (0,011) | 0,103*** (0,011) | 0,006 (0,012) | 0,008 (0,011) | -0,039* (0,018) | -0,047** (0,017) | -0,063*** (0,016) |
| Spielstätte | - | 0,241*** (0,035) | 0,261*** (0,032) | 0,133*** (0,037) | 0,155*** (0,039) | 0,151*** (0,036) | 0,286*** (0,032) |
| Aufführungen (in 100) | - | - | 0,817*** (0,084) | 0,669*** (0,087) | 0,685*** (0,089) | 0,698*** (0,091) | 0,008*** (0,001) |
| Subventionen (in 1 Mio. €) | - | - | - | 0,055*** (0,011) | 0,054*** (0,011) | 0,087*** (0,011) | - |
| Wettbewerb | - | - | - | - | 1,735*** (0,487) | 1,677*** (0,453) | 2,151*** (0,412) |
| Abonnements (in 1.000) | - | - | - | - | - | -0,025*** (0,003) | -0,016*** (0,003) |
| Privattheater | - | - | - | - | - | - | 0,165*** (0,035) |
| N | 418 | 418 | 418 | 418 | 415 | 402 | 405 |
| Prop > F | 0,000 | 0,000 | 0,000 | 0,000 | 0,000 | 0,000 | 0,000 |
| (adj) R-squared | 0,254 | 0,337 | 0,46 | 0,497 | 0,514 | 0,551 | 0,528 |
| RMSE | 1,874 | 1,769 | 1,598 | 1,546 | 1,521 | 1,467 | 1,502 |

*Anmerkung*: *p < 0,05, **p < 0,01, ***p < 0,001. Standardfehler in Klammern.

**Tabelle A7: OLS-Regression AV1 *Neuheit* mit fünfjährig gleitenden Mittelwerten mit geschätzten robusten Standardfehlern**

| **Variablen** | **(1)** | **(2)** | **(3)** | **(4)** | **(5)** | **(6)** | **(7)** |
| --- | --- | --- | --- | --- | --- | --- | --- |
| Anzahl der Stücke | 0,124*** (0,011) | 0,112*** (0,01) | 0,015 (0,011) | 0,019 (0,01) | -0,021 (0,017) | -0,031* (0,016) | -0,04* (0,018) |
| Spielstätte | - | 0,248*** (0,034) | 0,268*** (0,031) | 0,128*** (0,035) | 0,148*** (0,037) | 0,142*** (0,033) | 0,38*** (0,036) |
| Aufführungen (in 100) | - | - | 0,792*** (0,083) | 0,632*** (0,089) | 0,652*** (0,09) | 0,66*** (0,092) | 0,009*** (0,001) |
| Subventionen (in 1 Mio. €) | - | - | - | 0,057*** (0,01) | 0,057*** (0,01) | 0,092*** (0,011) | - |
| Wettbewerb | - | - | - | - | 1,393** (0,469) | 1,416** (0,43) | 1,757*** (0,461) |
| Abonnements (in 1.000) | - | - | - | - | - | -0,026*** (0,003) | -0,006 (0,003) |
| Privattheater |  |  |  |  |  |  | 0,114*** (0,023) |
| N | 380 | 380 | 380 | 375 | 375 | 366 | 371 |
| Prop > F | 0,000 | 0,000 | 0,000 | 0,000 | 0,000 | 0,000 | 0,000 |
| (adj) R-squared | 0,317 | 0,407 | 0,525 | 0,569 | 0,581 | 0,622 | 0,564 |
| RMSE | 1,717 | 1,601 | 1,435 | 1,373 | 1,356 | 1,29 | 1,382 |

*Anmerkung*: *p < 0,05, **p < 0,01, ***p < 0,001. Standardfehler in Klammern.

**Tabelle A8: OLS-Regression AV1 (ohne Köln) mit geschätzten robusten Standardfehlern**

| **Variablen** | **(1)** | **(2)** | **(3)** | **(4)** | **(5)** | **(6)** | **(7)** |
| --- | --- | --- | --- | --- | --- | --- | --- |
| Anzahl der Stücke | 0,086*** (0,012) | 0,079*** (0,012) | -0,028* (0,014) | -0,027* (0,014) | -0,11*** (0,02) | -0,113*** (0,019) | -0,128*** (0,019) |
| Spielstätte | - | 0,179*** (0,039) | 0,209*** (0,038) | 0,131** (0,049) | 0,178** (0,051) | 0,163** (0,052) | 0,254*** (0,044) |
| Aufführungen (in 100) | - | - | 0,946*** (0,105) | 0,849*** (0,109) | 0,877*** (0,107) | 0,875*** (0,108) | 0,888*** (0,115) |
| Subventionen (in 1 Mio. €) | - | - | - | 0,043** (0,016) | 0,035* (0,016) | 0,074*** (0,019) | - |
| Wettbewerb | - | - | - | - | 3,325*** (0,596) | 3,033*** (0,574) | 3,432*** (0,554) |
| Abonnements (in 1.000) | - | - | - | - | - | -0,023*** (0,004) | -0,014*** (0,003) |
| Privattheater | - | - | - | - | - | - | 0,244** (0,072) |
| N | 431 | 431 | 431 | 430 | 430 | 412 | 412 |
| Prop > F | 0,000 | 0,000 | 0,000 | 0,000 | 0,000 | 0,000 | 0,000 |
| (adj) R-squared | 0,129 | 0,168 | 0,321 | 0,333 | 0,383 | 0,404 | 0,403 |
| RMSE | 2,28 | 2,232 | 2,018 | 2,004 | 1,93 | 1,913 | 1,915 |

*Anmerkung*: *p < 0,05, **p < 0,01, ***p < 0,001. Standardfehler in Klammern.

**Tabelle A9: OLS-Regression AV1 (ohne Dinslaken) mit geschätzten robusten Standardfehlern**

| **Variablen** | **(1)** | **(2)** | **(3)** | **(4)** | **(5)** | **(6)** | **(7)** |
| --- | --- | --- | --- | --- | --- | --- | --- |
| Anzahl der Stücke | 0,083*** (0,012) | 0,074*** (0,012) | -0,045** (0,014) | -0,043** (0,014) | -0,13*** (0,02) | -0,129*** (0,019) | -0,144*** (0,019) |
| Spielstätte | - | 0,21*** (0,047) | 0,27*** (0,046) | 0,148** (0,051) | 0,188*** (0,052) | 0,169** (0,05) | 0,291*** (0,045) |
| Aufführungen (in 100) | - | - | 1,054*** (0,111) | 0,905*** (0,112) | 0,918*** (0,110) | 0,902*** (0,109) | 0,975*** (0,112) |
| Subventionen (in 1 Mio. €) | - | - | - | 0,058*** (0,014) | 0,058*** (0,013) | 0,089*** (0,015) | - |
| Wettbewerb | - | - | - | - | 3,428*** (0,593) | 3,242*** (0,571) | 3,723*** (0,55) |
| Abonnements (in 1.000) | - | - | - | - | - | -0,024*** (0,004) | -0,014*** (0,003) |
| Privattheater | - | - | - | - | - | - | 0,184*** (0,435) |
| N | 431 | 431 | 431 | 430 | 430 | 430 | 430 |
| Prop > F | 0,000 | 0,000 | 0,000 | 0,000 | 0,000 | 0,000 | 0,000 |
| (adj) R-squared | 0,108 | 0,152 | 0,315 | 0,35 | 0,402 | 0,434 | 0,491 |
| RMSE | 2,44 | 2,381 | 2,143 | 2,091 | 2,009 | 1,956 | 1,982 |

*Anmerkung*: *p < 0,05, **p < 0,01, ***p < 0,001. Standardfehler in Klammern.

**Tabelle A10: Fixed-Effects-Regression AV1 mit Driscoll-Kraay Standard Errors**

| **Variablen** | **(1)** | **(2)** | **(3)** | **(4)** | **(5)** | **(6)** | **(7)** |
| --- | --- | --- | --- | --- | --- | --- | --- |
| Anzahl der Stücke | -0,029 (0,017) | -0,035 (0,018) | -0,076*** (0,017) | -0,076** (0,017) | -0,486*** (0,039) | -0,488*** (0,037) | -0,487*** (0,03) |
| Spielstätte | - | 0,178** (0,061) | 0,217** (0,068) | 0,207** (0,066) | 0,203* (0,078) | 0,19* (0,074) | 0,216* (0,043) |
| Aufführungen (in 100) | - | - | 0,752*** (0,182) | 0,773** (0,201) | 0,672** (0,203) | 0,717** (0,203) | 0,677** (0,145) |
| Subventionen (in 1 Mio. €) | - | - | - | 0,041 (0,05) | 0,055 (0,031) | 0,089* (0,036) | - |
| Wettbewerb | - | - | - | - | 23,114*** (2,383) | 22,838*** (2,213) | 22,719*** (1,449) |
| Abonnements (in 1.000) | - | - | - | - | - | -0,023*** (0,005) | -0,016** (0,006) |
| Privattheater | - | - | - | - | - | - | -0,024 (0,064) |
| N | 454 | 454 | 454 | 453 | 453 | 435 | 435 |
| Prop > F | 0,103 | 0,015 | 0,000 | 0,000 | 0,000 | 0,000 | 0,000 |
| Within R-sqared | 0,008 | 0,033 | 0,071 | 0,073 | 0,425 | 0,444 | 0,435 |

*Anmerkung*: *p < 0,05, **p < 0,01, ***p < 0,001. Standardfehler in Klammern.

**Tabelle A11: OLS-Regression für Ur- oder Erstaufführungen (AV1) und Messung des Wettbewerbs in km mit geschätzten robusten Standardfehlern**

| **Variablen** | **(1)** | **(2)** | **(3)** | **(4)** | **(5)** | **(6)** | **(7)** |
| --- | --- | --- | --- | --- | --- | --- | --- |
| Anzahl der Stücke | 0,085*** (0,012) | 0,074*** (0,012) | -0,038** (0,014) | -0,037** (0,013) | -0,116*** (0,019) | -0,120*** (0,019) | -0,137*** (0,019) |
| Spielstätte | - | 0,23*** (0,043) | 0,255*** (0,04) | 0,129** (0,047) | 0,159** (0,048) | 0,155** (0,048) | 0,276*** (0,043) |
| Aufführungen (in 100) | - | - | 1,010*** (0,106) | 0,858*** (0,107) | 0,861*** (0,105) | 0,883*** (0,107) | 0,95*** (0,11) |
| Subventionen (in 1 Mio. €) | - | - | - | 0,058*** (0,014) | 0,059*** (0,013) | 0,089*** (0,015) | - |
| Wettbewerb | - | - | - | - | 8,402*** (1,506) | 7,84*** (1,453) | 9,311*** (1,387) |
| Abonnements (in 1.000) | - | - | - | - | - | -0,024*** (0,004) | -0,014*** (0,003) |
| Privattheater | - | - | - | - | - | - | 0,19*** (0,042) |
| N | 454 | 454 | 454 | 453 | 453 | 435 | 435 |
| Prop > F | 0,000 | 0,000 | 0,000 | 0,000 | 0,000 | 0,000 | 0,000 |
| (adj) R-squared | 0,111 | 0,171 | 0,326 | 0,361 | 0,408 | 0,432 | 0,419 |
| RMSE | 2,412 | 2,332 | 2,105 | 2,053 | 1,978 | 1,953 | 1,975 |

*Anmerkung*: *p < 0,05, **p < 0,01, ***p < 0,001. Standardfehler in Klammern.

**Tabelle A12: Poisson-Regression AV2 *Innovation* mit dreijährig gleitenden Mittelwerten**

| **Variablen** | **(1)** | **(2)** |
| --- | --- | --- |
| Stücke pro Jahr | -0,006* (0,003) | -0,007** (0,003) |
| Anzahl Neuheiten (AV1) | - | 0,319*** (0,063) |
| N | 260 | 258 |
| Prop > Chi² | 0,029 | 0,000 |
| LR Chi² | 4,77 | 28,00 |
| Pseudo R² | 0,018 | 0,107 |

*Anmerkung*: *p < 0,05, **p < 0,01, ***p < 0,001. Standardfehler in Klammern.

**Tabelle A13: Poisson-Regression AV2 *Innovation* mit fünfjährig gleitenden Mittelwerten**

| **Variablen** | **(1)** | **(2)** |
| --- | --- | --- |
| Stücke pro Jahr | -0,006* (0,003) | -0,008** (0,003) |
| Anzahl Neuheiten (AV1) | - | 0,296*** (0,064) |
| N | 260 | 260 |
| Prop > Chi² | 0,027 | 0,000 |
| LR Chi² | 4,91 | 23,87 |
| Pseudo R² | 0,02 | 0,095 |

*Anmerkung*: *p < 0,05, **p < 0,01, ***p < 0,001. Standardfehler in Klammern.

**Tabelle A14: Negative Binominal-Regression AV2 *Innovation***

| **Variablen** | **(1)** | **(2)** |
| --- | --- | --- |
| Stücke pro Jahr | -0,006* (0,003) | -0,006 (0,003) |
| Anzahl Neuheiten (AV1) | - | 0,343*** (0,05) |
| N | 260 | 254 |
| Prop > Chi² | 0,029 | 0,000 |
| LR Chi² | 4,78 | 46,57 |
| Pseudo R² | 0,016 | 0,158 |

*Anmerkung*: *p < 0,05, **p < 0,01, ***p < 0,001. Standardfehler in Klammern.
